# Cytoplasmic TDP-43 aggregation promotes oxidative stress and neurotoxicity through *Trx1* mRNA sequestration and translational suppression

**DOI:** 10.1016/j.redox.2026.104469

**Published:** 2026-11-04

**Authors:** Ji Min Lee, Eugene Bok, Hyeonjin Jeon, Seonhyeok Hwang, Jiwon Hong, Sung Bae Lee, Sun Joo Cha, Soo Hyun Kang, Hyung-Jun Kim, Mookyung Cheon, Byung-Hoon Lee, Chuna Kim, Sang-Won Lee, Sang Ryong Kim, Minyeop Nahm, Jaekwang Kim

**Affiliations:** a Dementia Research Group, Korea Brain Research Institute, Daegu, 41062, Republic of Korea; b School of Life Sciences and Biotechnology, BK21 FOUR KNU Creative BioResearch Group, Kyungpook National University, Daegu, 41566, Republic of Korea; c Aging Convergence Research Center, Korea Research Institute of Bioscience and Biotechnology, Daejeon, 34141, Republic of Korea; d Department of Chemistry, Center for Proteogenome Research, Korea University, Seoul, 02841, Republic of Korea; e Department of Brain Sciences, DGIST, Daegu, 42988, Republic of Korea; f Department of New Biology, DGIST, Daegu, 42988, Republic of Korea; g TargetX Inc., Seoul, 02841, Republic of Korea; h National Research Laboratory for Convergence Degradation Biology (NRLDB), Seoul, 02841, Republic of Korea; i Brain Science and Engineering Institute, Kyungpook National University, Daegu, 41944, Republic of Korea

**Keywords:** TDP-43 proteinopathy, Cytoplasmic aggregation, Thioredoxin 1, Oxidative stress, Mitochondrial dysfunction

## Abstract

Transactive response (TAR) DNA-binding protein 43 (TDP-43) is a multifunctional DNA/RNA binding protein. Cytoplasmic TDP-43 aggregation is a pathological hallmark of diverse neurodegenerative diseases collectively termed TDP-43 proteinopathy. Oxidative stress is closely associated with TDP-43 aggregation and neurotoxicity in TDP-43 proteinopathy, yet the underlying molecular mechanisms remain elusive. To delineate molecular basis of TDP-43 proteinopathy, we conducted deep quantitative proteomic profiling using a Neuro-2a (N2a) neuronal cell model of human wild-type TDP-43 overexpression (N2a-hTDP-43). We identified thioredoxin-1 (Trx1) as a central node linking mitochondrial dysfunction and redox dysregulation to TDP-43 pathology. Consistent with this, TDP-43 overexpression markedly reduced Trx1 protein levels. Moreover, inhibiting Trx1 exacerbates, whereas overexpressing Trx1 alleviates, cytoplasmic TDP-43 aggregation and neurotoxicity in N2a-hTDP-43 cells. Furthermore, overexpressing Trx1 alleviates TDP-43-associated pathologies in mouse primary neurons. Notably, we identified that cytoplasmic TDP-43 protein sequesters *Trx1* mRNAs into insoluble TDP-43 inclusions, leading to its translational suppression. We also demonstrated that suppression of Trx1 is a core mechanism eliciting oxidative stress and mitochondrial dysfunction in TDP-43 overexpression model. Importantly, in human induced pluripotent stem cell (iPSC)-derived motor neurons (MN) harboring the pathogenic TDP-43 M337V mutation (TDP-43^M337V^ iPSC-MNs), Trx1 expression is downregulated, concomitant with increased oxidative stress, compared to the isogenic TDP-43^WT^ iPSC-MN control. Furthermore, inducing Trx1 expression markedly reduces oxidative stress and cytoplasmic TDP-43 aggregation in TDP-43^M337V^ iPSC-MNs, suggesting conserved neuroprotective role of Trx1 in TDP-43 proteinopathy in human. Collectively, we identified Trx1 as a pivotal regulator of TDP-43 proteinopathy, highlighting therapeutic potential of Trx1 for TDP-43 proteinopathies.

## Highlights

• Trx1 is downregulated in TDP-43 proteinopathy.
• Cytoplasmic TDP-43 suppresses Trx1 translation by sequestering its mRNA.
• Inducing Trx1 attenuates TDP-43 proteinopathy.

## Graphical abstract

**Figure:**
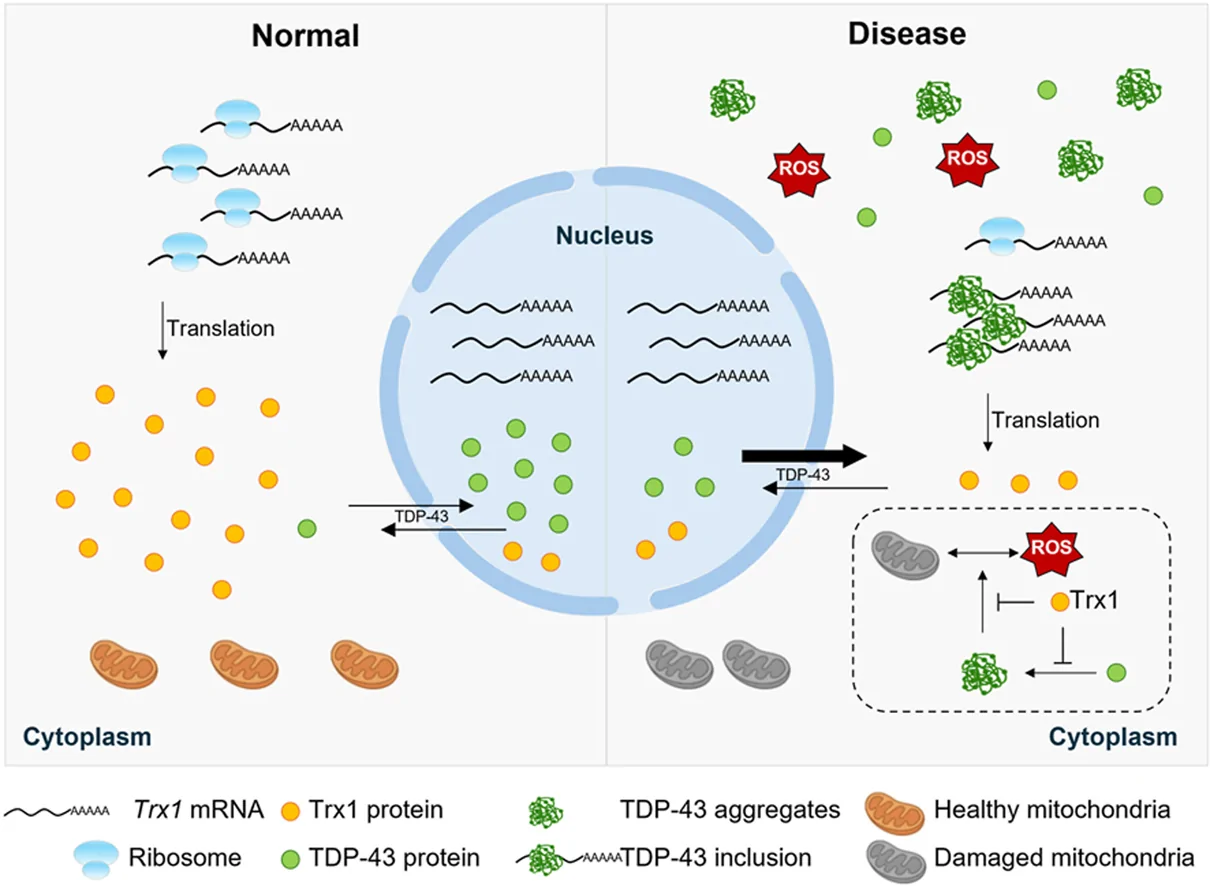

## 1 Introduction

TAR DNA-binding protein 43 (TDP-43) encoded by the *TARDBP* gene is evolutionally conserved among mammals and invertebrates and ubiquitously expressed in nearly all tissues [1], [2]. TDP-43 has been gaining growing attention as a key molecular player in various neurodegenerative diseases. A breakthrough in underpinning amyotrophic lateral sclerosis (ALS) pathogenesis is the discovery of TDP-43 as a major constituent of ubiquitin-positive inclusions in most cases of ALS (∼97 %) and in about 50 % of frontotemporal dementia (FTD) cases [3]. Various mutations in the *TARDBP* gene have been subsequently identified in both ALS and FTD, supporting the pathogenic importance of TDP-43 dysregulation in these diseases [4]. Beyond these, recent studies have identified TDP-43 inclusions in a wide range of neurodegenerative diseases collectively known as TDP-43 proteinopathies, including limbic predominant age-related TDP-43 encephalopathy (LATE), Alzheimer's disease (AD), Huntington's disease (HD), Parkinson's disease (PD), and traumatic brain injury (TBI) [5]. Currently, there is no cure for TDP-43 proteinopathies. To develop effective therapies for TDP-43-associated neurodegenerative diseases, a central question is how and to what extent TDP-43 dysregulation contributes to the onset and progression of each disease.

Although TDP-43 protein can shuttle between nucleus and cytoplasm, it is predominantly localized in the nucleus under normal physiological condition where it regulates RNA metabolism, such as transcription, mRNA splicing and polyadenylation [6], [7]. TDP-43 is also known to play a pivotal role in multiple cellular functions including regulation of stability, transport, translation of target mRNAs, and microRNA maturation [4], [5]. TDP-43 is also important for the protection of neurons against cytotoxic insults such as oxidative stress and regulation of stress granules [8], [9].

The cytoplasmic aggregation of TDP-43 is a key pathological hallmark of TDP-43 proteinopathy. TDP-43 mutations are not found in most cases of TDP-43 proteinopathies. However, under pathological settings, abnormal accumulation of wild-type TDP-43 (TDP-43 WT) induces cytoplasmic TDP-43 aggregation, leading to its depletion in the nucleus [2], [10]. These cytoplasmic TDP-43-positive inclusions are also observed in human induced pluripotent stem cell (iPSC)-derived neurons and glial cells expressing pathogenic mutant forms of TDP-43, such as TDP-43 Q331K and M337V [2], [11], [12]. Increasing evidence suggests that TDP-43 proteinopathy is driven by two non-mutually exclusive mechanisms: nuclear loss-of-function and cytoplasmic gain-of-function [2], [5], [10]. In line with the multiple functions of TDP-43, experimental evidence suggests that pathological dysregulation of TDP-43 induces neurodegeneration by interrupting multiple cellular pathways, such as RNA metabolism, proteostasis, oxidative stress responses, and mitochondrial function [4], [5]. Despite the expanding knowledge of TDP-43 pathophysiology [5], [6], [13], the detailed molecular mechanisms by which TDP-43 progressively disrupt neuronal functionality remain elusive.

A remaining key question is what core mechanism triggers and/or mediates TDP-43 proteotoxicity leading to neuronal death. It is noteworthy that mitochondrial dysfunction and oxidative stress are closely associated with TDP-43 toxicity. Many studies have commonly reported increased oxidative stress in the postmortem neuronal tissues of ALS and other TDP-43 proteinopathies [14], [15]. However, it remains elusive whether oxidative stress is a primary cause or a secondary consequence, and whether it is initiated in an early or late stage of pathology in humans due to the lack of reliable biomarkers for the early preclinical stage. However, experimental evidence from preclinical models of TDP-43 proteinopathies suggests that oxidative stress can trigger cytoplasmic TDP-43 aggregation in neuronal cells [16], [17], which is recapitulated in human patient fibroblasts and iPSC-derived motor neurons (MN) [18]. In addition, mutant TDP-43 impairs the cellular antioxidant defense system and increases oxidative stress in neuronal cells [19]. Moreover, inhibiting oxidative stress using antioxidants has been proven to be protective against TDP-43-mediated neurotoxicity [15], [20], [21]. Taken together, increasing evidence indicates that oxidative stress plays a pivotal role in TDP-43 proteinopathy both as an upstream driver and a downstream effector of TDP-43-associated neurotoxicity. A central yet unresolved question is what molecular mechanisms drive oxidative stress in TDP-43 proteinopathy.

To answer this question, we aimed to comprehensively profile the proteomic changes in TDP-43 proteinopathy using a N2a neuronal cell model of human wild-type TDP-43 overexpression (N2a-hTDP-43), which recapitulates the key pathological features of TDP-43 proteinopathy, such as cytoplasmic TDP-43 aggregation, oxidative stress, mitochondrial dysfunction, and neurotoxicity. Proteomic profiling identified thioredoxin 1 (Trx1), a master regulator of redox homeostasis, as a pivotal regulator of TDP-43 pathology. We revealed that inhibiting Trx1 aggravates TDP-43 pathology, whereas overexpressing Trx1 markedly mitigates TDP-43 pathology in N2a-hTDP-43 cells. We further demonstrated that cytoplasmic TDP-43 protein sequesters *Trx1* mRNA into insoluble TDP-43 inclusions, thereby suppressing its translation. In addition, overexpressing Trx1 strongly alleviates TDP-43-associated neurotoxicity in mouse primary cortical neurons. Furthermore, Trx1 protein levels are downregulated in human iPSC-MNs harboring the TDP-43 M337V mutation (TDP-43^M337V^ iPSC-MNs) compared to isogenic TDP-43^WT^ iPSC-MNs. Moreover, overexpressing Trx1 significantly suppresses oxidative stress and cytoplasmic TDP-43 aggregation in TDP-43^M337V^ iPSC-MNs. Taken together, our results indicate that cytoplasmic TDP-43 aggregation promotes oxidative stress and neurotoxicity by sequestering *Trx1* mRNA, thereby suppressing its translation.

## 2 Materials and methods

### 2.1 Plasmid and virus preparation

The coding sequences of full-length human wild-type *TDP-43* (*hTDP-43*; NM_007375) and C-terminal FLAG-tagged *hTDP-43* (*hTDP-*43-FLAG) were amplified from the pCMV6-AC-hTDP-43 WT-GFP plasmid (OriGene, RG210639). The C-terminal FLAG-tagged hTDP-43-5FL mutant, in which phenylalanine residues F147, F149, F194, F229, and F231 were substituted with leucine [22], [23], was synthesized by Bioneer (Korea). All hTDP-43 inserts were cloned into the pAAV-MCS expression vector using the *Bam*HI and *Xho*I restriction sites. The coding sequences of full-length human wild-type thioredoxin 1 (*hTrx1*; NM_003329) with a C-terminal Myc-FLAG tag was amplified from pCMV6-hTrx1-Myc-FLAG (OriGene, RC208876) and cloned into the pAAV-MCS expression vector using the *Bam*HI and *Xba*I restriction sites. The EGFP coding sequence was subcloned into the pAAV-MCS expression vector using the *Eco*RI and *Bam*HI restriction sites to generate the pAAV-EGFP benign control vector. The plasmid encoding human ribosomal protein L22 with a C-terminal Myc-FLAG tag (hRPL22-Myc-FLAG; NM_000983) was obtained from OriGene (RC208910). ON-TARGETplus mouse Trx1 siRNA SMARTpool (siTrx1; Dharmacon, L-062799-01-0005) targeting endogenous mouse Trx1 and ON-TARGETplus Non-targeting control siRNA (siCon; Dharmacon, D-001810-10-05) were purchased from Dharmacon. The empty pAAV-MCS (pAAV-Con), pAAV-EGFP, pAAV-hTDP-43, and pAAV-hTrx1-Myc-FLAG were packaged into serotype 8 capsid through VectorBuilder (https://www.vectorbuilder.kr/) to generate AAV-Control, AAV-EGFP, AAV-hTDP-43, and AAV-hTrx1 viruses respectively.

### 2.2 Cell culture and transfection

N2a mouse neuroblastoma cells (CCL-131, ATCC) were plated at a density of 2 × 10^5^ cells/mL and grown in Dulbecco's modified Eagle's medium (DMEM) supplemented with 10 % fetal bovine serum (FBS) and 1 % penicillin/streptomycin (P/S) at 37 °C, in a humidified 5 % CO_2_ incubator. Plasmids were transfected into cells using Lipofectamine 2000 (11668-019, Invitrogen) as indicated according to the manufacturer's instructions. Corresponding empty plasmids (Con) or EGFP-expressing plasmids (EGFP) were used as negative controls. For knockdown experiments, N2a cells were transfected with Trx1 siRNA (siTrx1, L-062799-01-0005, Dharmacon) using Lipofectamine RNAiMAX (13778075, Invitrogen) according to the manufacturer's instructions. Non-targeting control siRNAs (siCon, D-001810-10-05, Dharmacon) were used as a negative control. For stress granule induction, cells were treated with 0.5 mM sodium arsenite (NaAsO_2_) for 30 min prior to fixation.

### 2.3 Primary cortical neuron culture

All experiments with animals were approved by the Institutional Animal Care and Use Committee (IACUC) at Korea Brain Research Institute (IACUC-23-00006). C57BL/6 mice were purchased from KOATECH, Korea. Primary cortical neurons were isolated from embryonic day 16 (E16) mouse embryos. The cerebral cortices were dissected in Leibovitz's L-15 medium (LM003-01, Welgene) and digested with EBSS supplemented with 20 units/mL papain and 2000 units/mL DNase I (LK003153, Worthington Biochemical Corporation) for 20 min at 37 °C with gentle agitation. The tissues were then gently triturated with a pipette and filtered through a 40 μm nylon cell strainer to obtain a single-cell suspension. Cells were pelleted by centrifugation at 783 × g for 10 min at room temperature (RT). The pellets were resuspended with 10 mg/mL ovomucoid protease inhibitor containing DNase I 2000 units/mL (Worthington Biochemical Corporation) and centrifuged again at 783 × g for 10 min at RT. Subsequently, cells were resuspended in Neurobasal medium supplemented with 2 % B-27, 0.5 mM GlutaMAX, and 1 % P/S and plated at a density of 3 × 10^5^ cells/mL on poly-d-lysine (PDL, LK003153, Sigma-Aldrich)-coated culture plates or glass coverslips. Neurons were maintained at 37 °C in a humidified 5 % CO_2_ incubator, and one-third of the culture medium was replaced every 3–4 days. To overexpress hTDP-43 and hTrx1, neurons were transduced at 7 days in vitro (DIV7) with AAV-hTDP-43 and/or AAV-hTrx1-Myc-FLAG viruses (5 × 10^7^ GC/μL). AAV-Con or AAV-EGFP virus were used as a negative control. The total viral titer used for transduction was kept constant across all groups.

### 2.4 Differentiation of iPSCs to motor neurons

The control iPSC line, KOLF2.1 J (JIPSC1000) was obtained from The Jackson Laboratory. iPSCs were cultured in StemFlex™ medium, consisting of StemFlex™ Basal Medium and StemFlex™ supplement (A3349401, Gibco) in Matrigel (354277, Corning)-coated plates for 3–5 days. Following detachment with Accutase solution (A1110501, Gibco), embryoid bodies (EBs) were generated in ultra-low attachment plates (7007, Corning). On day 0, the culture medium was replaced with EB medium, consisting of a 1:1 mixture of Advanced DMEM-F12 and Neurobasal, supplemented with 1 × N2, 2 % B-27, 0.5 mM GlutaMAX, 100 μM 2-mercaptoethanol, 1 % P/S, 10 ng/mL bFGF, 10 μM Y-27632 dihydrochloride, 10 μM SB431542, 3 μM CHIR99021, 100 nM LDN193189, and 30 μg/mL ascorbic acid. From day 2 to day 6, the medium was additionally supplemented with 100 nM retinoic acid, 500 nM purmorphamine, and 500 nM smoothened agonist (SAG; Merck). During this period, Y-27632 dihydrochloride and bFGF were removed. On day 7, 10 ng/mL BDNF was added, while SB431542, CHIR99021, and LDN193189 were withdrawn. From day 9 to day 13, 10 μM DAPT was added. On day 14, 10 ng/mL GDNF was added, and DAPT was removed. The medium was half changed every 2–3 days. On day 16, EBs were enzymatically dissociated into single cells using 0.05 % trypsin and filtered through a 40 μm cell strainer. Cells were counted and seeded onto Laminin/Poly-l-Ornithine-coated plates. The MN medium consisted of Neurobasal, 1X non-essential amino acids, 0.5 mM GlutaMAX, 100 μM 2-mercaptoethanol, and 1 % P/S. This medium was further supplemented with 10 ng/mL GDNF, 10 ng/mL BDNF, 10 ng/mL CNTF, 10 ng/mL IGF-1, 100 nM retinoic acid, 30 μg/mL ascorbic acid, 1X N2, and 2 % B-27. On the following day, cells were cultured in MN medium supplemented with 5 μM aphidicolin. After 24 h, the medium was replaced with fresh MN medium without aphidicolin. MNs were maintained under differentiation conditions until experimental analysis. For hTRX1 overexpression, MNs were transduced with AAV-hTrx1-myc-FLAG (1 × 10^8^ GC/μL) on day 30 and maintained for 7 days to ensure transduction efficiency. Cells were then fixed and processed for immunocytochemistry.

### 2.5 Proteomics

N2a cells were plated in a 100 mm dish at a density of 2.1 × 10^6^ cells/dish. After 24 h, cells were transfected with empty pAAV (pAAV-Con) or pAAV-hTDP-43 WT plasmid using Lipofectamine 2000 reagent. 24 h post-transfection, cells were harvested. For all samples, proteins were extracted using an SDS-based lysis buffer (1 % SDS with phosphatase inhibitor (PhosSTOP, Roche), in 0.1 M Tris-HCl, pH 7.4) and sonicated. Protein lysates were digested by applying a modified filter-aided sample preparation (FASP) method. Briefly, proteins lysates were reduced with 100 mM dithiothreitol (DTT), boiled and centrifuged to remove debris, and the supernatants were transferred to membrane filters (YM-30, Millipore Corporation). Transferred samples were washed with 8 M urea Tris-HCl (pH 8.5), then alkylated with 50 mM iodoacetamide (IAA) and incubated in dark. Additional wash was performed with 8 M urea, then buffer was exchanged to 50 mM ammonium bicarbonate (ABC) prior to digestion. Collection tubes were exchanged, and digestion was performed by adding trypsin (V5111, Promega) to the samples with an enzyme to protein ratio (w/w) of 1:50 for the first digestion (12 h) and 1:100 for the second digestion (6 h) both incubated at 37 °C. Elution of peptides was performed by centrifugation and addition of 50 mM ABC. Peptide concentrations were determined using a BCA assay, and the quantified peptides were aliquoted into 100 μg aliquots then vacuum dried for subsequent TMT labelling. A universal reference was generated by pooling 50 μg of all sample peptides and aliquoted into 100 μg units.

Two 18-plex TMTpro sets were prepared, each including two biological replicates per condition and two identical universal reference channels. Two replicates of 24 h pAAV-Con were labeled to channels 127 N and 131 N, those for 24 h pAAV-hTDP-43 WT were labeled to channels 127C and 131C in each TMT set. 126 and 135 N channels were labeled with universal references. A total of 3.6 mg of TMT peptides (i.e., 200 μg of peptides per channel) from each experimental set were labeled with two units of TMTpro reagent (A44522 and A52046, Thermo Fisher Scientific) according to the manufacturer's instructions. Labeled peptides were desalted using an Agilent 1260 Infinity HPLC (Agilent). Collected peptides were dried, and 50 μg was subjected to global proteomics profiling.

For LC-MS/MS analysis, a previously developed 24-fraction dual online non-contiguous fractionating and concatenating reverse phase/reverse phase LC (DO-NCFC-RP/RPLC) system was coupled with an Orbitrap Exploris 480 mass spectrometer (Thermo Fisher Scientific) via an in-house dual nanoelectrospray source [24]. Injected samples were fractionated online into 24 fractions at mid-pH, with each fraction individually analyzed using an in-house-manufactured analytical and SPE columns packed with Jupiter C18 particles (3 μm, 300 Å, Phenomenex). For each analysis, an 180 min gradient was applied with solvent B (0.1 % FA in ACN) increasing from 10 % to 37.5 % and solvent A consisting of 0.1 % FA in water. As for MS parameters, the isolation window was set to 0.7 Th with other parameters set as previously described [24].

Each MS/MS dataset was subjected to multiplexed post-experimental monoisotopic mass refinement method (mPE-MMR) [25]. Resultant mgf files were searched against a uniprot mouse protein database (released November 2021; 63,639 entries) including contaminants (179 entries) using MS-GF + search engine (v9949) and global search parameters set as previously described, with an addition of static modification of TMTpro (+304.207146 Da) on N termini and lysine instead of TMT [26]. Search results from both TMT sets were combined and filtered at a false discovery rate (FDR) of 1 % at peptide-spectrum match (PSM) level. Proteins were inferred from the identified peptides, and the protein supported by the largest number of peptides within each protein group was selected as its representative protein.

### 2.6 Proteomics data analysis

The identified peptides with PSM-level FDR < 0.01 from two 18-plex TMT datasets were combined to generate an alignment table in which the identified peptides from different TMT datasets with the same sequences were matched. Of the identified peptides, we first selected the unique peptides that were detected in both TMT experiments. The reporter ion intensities of these selected unique peptides in the alignment table were normalized by the intensities of the universal reference samples in the corresponding TMT experiment to remove the batch effects of each TMT experiment. Normalized intensity values were used as input for PCA and heatmap plotting, with NaN values removed prior to statistical evaluation. PCA was performed using the R package FactoMineR (v2.11), and visualization was carried out with factoextra (v1.0.7).

To identify differentially expressed proteins (DEPs), we first selected a set of peptides whose abundances were detected in both TMT experimental sets to ensure their statistical power. We then performed a Student's *t*-test for these peptides. We selected differentially expressed peptides with a *p*-value ≤ 0.05 and ratio ≥ 1.5 and then identified differentially expressed proteins containing the differentially expressed peptides. For visualization, the intensity values of DEPs were used to generate group-wise heatmaps with the R package pheatmap (v1.0.12).

Overrepresentation analysis was performed using the R package clusterProfiler (v4.10.1) with the C5 Gene Ontology (GO) collection from MSigDB (v2023.2). Human gene sets were converted to mouse orthologs and filtered for valid mappings. Significant up- and down-regulated DEP lists were analyzed separately. Enriched pathways with *p*-value < 0.05 were visualized using ggplot2 (v3.5.1).

Enrichment results were visualized and clustered in Cytoscape (v3.10.3) using EnrichmentMap (v3.5.0) and AutoAnnotate (v1.5.2). Gene-set similarity edges were obtained by applying the AutoAnnotate option ‘Amount of clusters’ at level 3. Venn diagrams illustrating the overlap of DEPs among the four major modules of the downregulated Cytoscape network were generated using the R package ggvenn (v0.1.10). PPI networks were generated by using a stringApp (v2.2.0) with a confidence score cutoff of 0.4 using the protein list from selected submodules.

### 2.7 Immunocytochemistry

Cells were fixed with 4 % paraformaldehyde (PFA) for 15 min at RT. The cells were rinsed with PBS and permeabilized with PBS containing 0.2 % Triton X-100 for 15 min. After blocking with 3 % BSA (Sigma-Aldrich) for 30 min, cells were incubated overnight at 4 °C with primary antibodies diluted in PBS containing 3 % BSA. The following primary antibodies were used: *anti*-TDP-43 (80001-1-RR, 80002-1-RR, Proteintech), *anti*-Trx1 (14999-1-AP, Proteintech), anti-cleaved caspase-3 (9661, Cell Signaling Technology), *anti*-Myc (ab43, Abcam), *anti*-TUBB3 (5568S, Cell Signaling Technology; 802001, Biolegend), *anti*-TIA1 (610352, BD Transduction), *anti*-G3BP1 (611127, Waters Biosciences), *anti*-NeuN (MAB377, Millipore), *anti*-ISL-1/2 (39.4D5, DSHB), *anti*-HB9 (81.5C10, DSHB). Cells were rinsed three times with PBS and then incubated for 1 h at RT with secondary antibodies, including Alexa Fluor® 488 (715-545-151, Jackson ImmunoResearch) or Alexa Fluor® 594 AffiniPure Donkey Anti-Mouse IgG (H + L) (715-585-150, Jackson ImmunoResearch), Alexa Fluor® 488 (715-545-152, Jackson ImmunoResearch) or Alexa Fluor® 594 AffiniPure Donkey Anti-Rabbit IgG (H + L) (711-585-152, Jackson ImmunoResearch), Alexa Fluor® 488 goat anti-Rabbit IgG (H + L) (A11008, Invitrogen), or Alexa Fluor® 555 goat anti-Mouse IgG (H + L) (A21422, Invitrogen). Nuclei were counterstained with DAPI (D9542, Sigma-Aldrich), and coverslips were mounted using VECTASHIELD Antifade Mounting Medium (H-1000, Vector Laboratories). Stained cells were imaged using a confocal microscope (Leica, TCS SP8 and STELLARIS 8) and analyzed using MetaMorph (Molecular Devices) and ImageJ software.

### 2.8 Measurement of ROS levels

Intracellular reactive oxygen species (ROS) levels were measured using the reagent (C10422, Thermo Fisher Scientific) according to the manufacturer's instructions. Briefly, cells were incubated with 5 μM CellROX™ Deep Red in maintenance medium for 30 min at 37 °C in a humidified 5 % CO_2_ incubator. Subsequently, cells were washed with PBS to remove excess dye and then fixed with 4 % PFA for 15 min at RT. Nuclei were counterstained with DAPI. After mounting, cells were imaged using a confocal microscope (Leica, TCS SP8 and STELLARIS 8) and analyzed using ImageJ software.

### 2.9 Mitochondrial bioenergetics measurement

Oxygen consumption rate (OCR) was measured using a Seahorse XF Analyzer (Agilent Technologies) as described previously [20]. Briefly, N2a cells were seeded in Seahorse XF cell culture microplates at a density of 6 × 10^4^ cells/well and incubated overnight 37 °C in a humidified 5 % CO_2_ incubator. 24 h post-plating, cells were transfected with hTDP-43 and/or hTrx1 plasmids. 48 h post-transfection, OCR was measured using a Seahorse XF Analyzer. The day before the assay, the sensor cartridge was hydrated overnight at 37 °C in a non-CO_2_ incubator using Seahorse XF calibrant solution for proper calibration. Prior to measurement, the culture media were replaced with Seahorse XF assay medium (RPMI-based, pH 7.4) supplemented with 10 mM glucose, 1 mM pyruvate, and 2 mM GlutaMAX, and cells were degassed for 1 h in a non-CO_2_ incubator. The OCR was measured at baseline, followed by sequential additions of 2 μM oligomycin, 2 μM FCCP, and a mixture of rotenone and antimycin A (0.5 μM each). OCR values were collected using Wave software (Agilent Technologies) and calculated according to the manufacturer's protocol. All OCR values were normalized to the corresponding protein levels.

### 2.10 Immunoprecipitation

At 48 h post-transfection, N2a cells were harvested and lysed in ice-cold lysis buffer containing 25 mM Tris-HCl (pH 8.0), 150 mM NaCl, 2 mM MgCl_2_, 0.5 % NP-40, and 1× EDTA-free protease inhibitor cocktail for 30 min on ice. For RNA immunoprecipitation (RIP) experiments, lysis buffer was supplemented with 250 U/mL RNasin (Promega). Cell lysates were centrifuged at 14,000 × g for 15 min and supernatants were collected. The protein concentration of cell lysates was measured using the Pierce™ BCA Protein Assay Kit (23225, Thermo Fisher Scientific). Lysates were clarified by centrifugation at 14,000 × g for 20 min at 4 °C, and total protein concentrations were determined using the BCA Protein Assay Kit. For immunoprecipitation of Flag-tagged proteins, 1 mg of total protein was incubated with Pierce™ *Anti*-DYKDDDDK Magnetic Agarose beads (A36797, Thermo Fisher Scientific) overnight at 4 °C with gentle rotation. Beads were washed three times with lysis buffer to remove nonspecific binding. For RIP, an additional wash was performed using lysis buffer containing 0.04 U/mL DNase I for 10 min at 37 °C. Proteins were eluted by incubating the beads with 1.5 mg/mL FLAG peptide (A36805, Thermo Fisher Scientific) for 10 min at RT. The input and eluted samples were analyzed by Western blot. For RNA analysis, the RNAs bound to immunoprecipitated proteins were purified using Quick-RNA Microprep Kit (R1051, Zymo Research) according to the manufacturer's instructions.

### 2.11 Westen blot analysis

For total protein extraction, cells were lysed in RIPA buffer (2.5 mM Tris-HCl, pH 7.4, 1 % NP-40, 0.25 % deoxycholic acid, 150 mM NaCl, 1 mM EDTA, 0.1 % SDS) supplemented with 1X protease inhibitor cocktail (Roche). For Triton X-100 fractionation, cells were lysed in Triton X-100 lysis buffer (1 % Triton X-100, 50 mM Tris-HCl, pH 7.4, 150 mM NaCl, 1 mM EDTA, 1X protease inhibitor cocktail) for 20 min on ice. Lysates were centrifuged at 100,000 × g for 30 min at 4 °C, and the supernatant was collected as the Triton X-100–soluble fraction. The resulting pellets were washed with lysis buffer by centrifugation at 100,000 × g for 30 min at 4 °C. The pellets were then resuspended in 1 % SDS lysis buffer (1 % SDS, 10 mM Tris-HCl, pH 7.4, 1X protease inhibitor cocktail) for 30 min at RT and sonicated (50 % amplitude, 1 s ON/2 s OFF, 20 cycles). Total protein concentrations were determined using the BCA Protein Assay Kit (23225, Thermo Fisher Scientific). Protein lysates were boiled for 10 min at 95 °C in SDS sample buffer (62.5 mM Tris-HCl, pH 6.8, 10 % glycerol, 2 % SDS, 20 % β-mercaptoethanol, and 0.005 % bromophenol blue). Equal amounts of proteins were then separated by SDS-PAGE and transferred onto 0.2 μm nitrocellulose (1620112, Bio-Rad) or polyvinylidene fluoride membranes (IPVH00010, Millipore). The membranes were then blocked with 4 % non-fat dry milk or BSA in Tris-buffered saline containing 0.125 % Tween-20 (TBS/T) for 20 min at RT. Blots were probed in blocking buffer with *anti*-TDP-43 for human TDP-43 (H00023435-M01, Novus Biologicals), *anti*-TDP-43 for both human and mouse TDP-43 (80001-1-RR, Proteintech), *anti*-Trx1 (14999-1-AP, Proteintech), anti-FLAG (F3165, Sigma-Aldrich), anti-Histone H3 (9715, Cell Signaling Technology), *anti*-Myc (2272S, Cell Signaling Technology), anti-cleaved caspase-3 (9661, Cell Signaling Technology), anti-pro-caspase-3 (9662, Cell Signaling Technology), *anti*-Hsp70 (sc-32239, Santa Cruz), or *anti*-GAPDH (10494-1-AP, Proteintech) for 2 h at RT. After secondary antibody incubation at RT for 1 h, blots were developed using Lumigen ECL Ultra (TMA-100, Lumigen, Southfield, Michigan, USA) and imaged with the ImageQuant 800 system (Amersham). The levels of protein were quantified using Quantity One 4.6.6 (Basic) and normalized as indicated.

### 2.12 Quantitative real time polymerase chain reaction (qRT-PCR)

Total mRNAs were extracted using TRI Reagent (TR 118, Molecular Research Center) or the Quick-RNA Microprep Kit (R1051, Zymo Research) according to the manufacturers' instructions. For subcellular fractionation, cells were homogenized in ice-cold low-salt buffer (10 mM Tris-HCl, pH 7.5, 5 mM EDTA, 10 % sucrose) supplemented with 250 U/mL RNasin (Promega, Madison, WI, USA) and 1X protease inhibitor cocktail using a Teflon homogenizer. To ensure complete mechanical disruption, cells were subsequently passed through a 27-gauge needle 20 times. One-fifth of the total homogenates was saved as the total fractions. The remaining homogenates were centrifuged at 700 × g for 20 min at 4 °C to separate nuclei (pellet) from cytoplasm (supernatant). Cytoplasmic fractions were supplemented with 1 % Triton X-100, incubated on ice for 30 min, and centrifuged at 100,000 × g for 30 min at 4 °C to obtain Triton X-100-soluble and -insoluble fractions. mRNAs were purified using the Quick-RNA Microprep Kit (Zymo Research) according to the manufacturer's instructions. Complementary DNAs (cDNAs) were synthesized from the extracted RNA using the High-Capacity cDNA Reverse Transcription Kit (4374966, Applied Biosystems). Quantitative PCR was performed with Fast SYBR™ Green Master Mix (4385616, Applied Biosystems) using the default thermal cycling program in QUANTSTUDIO5 (Applied Biosystems). Relative levels of mRNAs were calculated using the comparative *C*_t_ method using GenEX 6 and normalized as indicated. The levels of mRNAs were measured using a primer set for each gene (Table 1).

**Table 1:** Primers for qRT-PCR.

Gene	Forward Primer (5′ to 3′)	Reverse Primer (5′ to 3′)
*mTDP-43*	CCTTTGCAGATGATAAGGTTGCC	TGTGCAGCGTGATGACGAA
*hTDP-43*	GGGTAACCGAAGATGAGAACG	CTGGGCTGTAACCGTGGAG
*mTrx1*	CATGCCGACCTTCCAGTTTTA	TTTCCTTGTTAGCACCGGAGA
*Gapdh*	AGGTCGGTGTGAACGGATTTG	TGTAGACCATGTAGTTGAGGTCA

### 2.13 Proximity ligation assay (PLA)

Proximity ligation assay (PLA) was performed using the PLA Kit (DUO92101, Sigma-Aldrich) according to the manufacturer's instructions. Cells were fixed with 4 % PFA for 15 min at RT, rinsed with PBS, and permeabilized with PBS containing 0.2 % Triton X-100 for 15 min. After blocking with Duolink® Blocking Solution for 1 h at 37 °C, cells were incubated overnight at 4 °C with primary antibodies diluted in Duolink® Antibody Diluent. The following primary antibodies were used: *anti*-TDP-43 (80001-1-RR, Proteintech), *anti*-Trx1 (14999-1-AP, Proteintech), *anti*-Hsp70 (SC-32239, Santa Cruz) and anti-FLAG (F3165, Sigma-Aldrich). After washing with Wash Buffer A, cells were incubated with species-specific PLA PLUS and MINUS probes for 1 h at 37 °C. After washing, ligation was performed using the Duolink Ligase solution for 30 min at 37 °C, followed by rolling circle amplification using the Duolink Polymerase solution for 100 min at 37 °C according to the manufacturer's instructions. Samples were subsequently washed and mounted using Duolink In Situ Mounting Medium with DAPI (Sigma-Aldrich). PLA signals were visualized using a confocal microscope (Leica, STELLARIS 8).

### 2.14 Statistical analysis

Statistical analyses were performed using two-tailed Student's *t*-test or one-way ANOVA as indicated depending on comparison variables as indicated using GraphPad Prism 8. All data are shown as mean ± SEM. A *p*-value < 0.05 was considered statistically significant. The statistical methods and sample size are indicated in the figure legends.

## 3 Results

### 3.1 Proteomic profiling reveals Trx1 as a pivotal regulator of TDP-43 pathology

To unravel the molecular details underlying TDP-43 proteinopathy, we developed a N2a neuronal cell model of TDP-43 overexpression (N2a-hTDP-43). Overexpression of full-length human wild-type TDP-43 (hTDP-43) increased total TDP-43 protein levels to approximately twofold over endogenous TDP-43 protein levels in N2a-hTDP-43 cells (Fig. S1A and B). Our model recapitulates the key pathological features of TDP-43 proteinopathy, including cytoplasmic TDP-43 aggregation (Fig. S1C and D) and oxidative stress (Fig. S1E), without detectable stress granule formation (Fig. S1F and G). We next conducted deep quantitative profiling of the whole proteome by comparing control (N2a-Con) and N2a-hTDP-43 cells (Fig. 1A). Principal component analysis (PCA) shows a clear separation between control and N2a-hTDP-43 groups (Fig. 1B). Differential expression analysis identified 534 upregulated and 427 downregulated proteins, and a heat map of normalized protein intensities showed marked differences between groups (Fig. 1C). Gene Ontology Biological Process (GO:BP) overrepresentation analysis, retaining the top 20 enriched terms per direction, showed upregulation of ribosome biogenesis, rRNA processing, ribonucleoprotein complex assembly, and Golgi organization/ER–to–Golgi transport, whereas downregulated proteins were enriched for RNA processing/splicing, RNA transport/localization, mitochondrial organization, and protein folding (*p* < 0.05) (Fig. 1D).

**Fig. 1:**
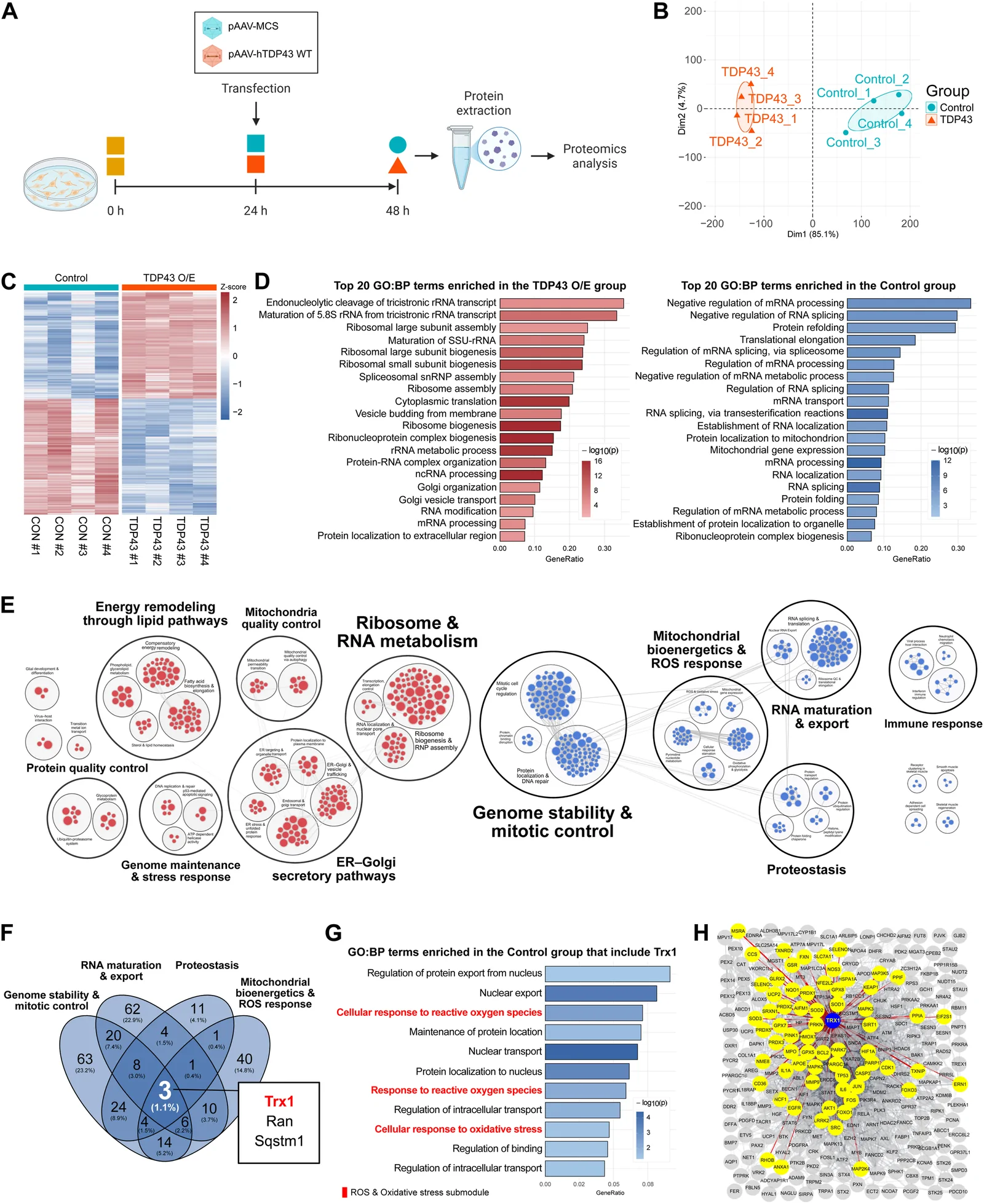
**Proteomic profiling in a N2a model of TDP-43 overexpression.** (A) Schematic diagram of proteomic profiling. 24 h post-plating, N2a cells were transfected with either hTDP-43 or control. 24 h post-transfection, proteins were extracted for subsequent proteomic analyses. (B) Principal component analysis (PCA) score plot. Samples were projected onto a two-dimensional plane defined by the first two principal components (PC1 and PC2). Percentages in parentheses indicate the variance explained by each component. (C) Heatmap of normalized intensities of DEPs with statistical significance. (D) Bar plot representing enriched GO:BP terms identified by overrepresentation analysis (ORA) of significantly up-and down-regulated DEPs. Bar length indicates the GeneRatio of DEPs within each pathway, while bar color reflects the–log_10_ (*p*-value) gradient. (E) Cytoscape network visualization of GO:BP ORA results, including pathways with *p* < 0.05. AutoAnnotate clustering was applied to group related pathways. The size of the node is proportional to the number of DEPs identified in each pathway gene set. (F) The Venn diagram presents the overlap among DEPs across the four major clusters of the downregulated Cytoscape network. (G) Bar plot of down-regulated GO:BP ORA pathways that include Trx1. Pathways highlighted in red correspond to the ROS and Oxidative stress submodule. (H) Protein–protein interaction network of proteins constituting the ROS and Oxidative stress submodule (A confidence score cutoff of 0.4). Trx1 exhibited extensive interactions with other proteins in the cluster (red).

To account for overlap among GO terms and reveal relationships among enriched categories, we generated a Cytoscape enrichment map (Fig. 1E). In the upregulated modules, we observed ribosome RNA metabolism, ER–Golgi trafficking defects previously reported in association with TDP-43 [27], as well as the generation of alternative energy pathways likely reflecting mitochondrial dysfunction (Fig. 1E). In contrast, the downregulated modules comprised processes critical for cellular homeostasis, including genome stability, RNA maturation, and proteostasis, as well as mitochondrial bioenergetics with ROS response (Fig. 1E). These data indicate that our model faithfully recapitulates the molecular alterations associated with TDP-43 proteinopathy, as previously reported [2], [4], [6], [27]. To identify key players shared across these downregulated modules, we intersected proteins assigned to these modules and found three recurrent proteins, including Trx1, RAN, and SQSTM1 (p62) (Fig. 1F). Trx1 is mapped to the ROS/oxidative stress module (Fig. 1G). Among the down-regulated pathways that include Trx1, GO:BP terms involving response to oxidative stress and protein/RNA transport were significantly enriched (*p* < 0.05) (Fig. 1G). A STRING protein–protein interaction network showed Trx1 connected to multiple members of the ROS and Oxidative stress module (Fig. 1H). Together, our data indicate coordinated perturbation of mitochondrial bioenergetics and redox homeostasis, with Trx1 positioned as a potential hub molecule within the affected networks. Trx1 is a major antioxidant system along with glutathione antioxidant system in mammalian cells [28]. As aforementioned, oxidative stress plays a pivotal role in the TDP-43-associated pathogenesis. Therefore, we sought to further delineate the role of Trx1 in TDP-43 proteinopathy.

### 3.2 Trx1 is protective against TDP-43 pathology in neuronal cells

To validate our proteomic data, we first assessed the alteration of Trx1 at the protein level upon TDP-43 overexpression by immunostaining with Trx1-specific antibody in N2a cells. The specificity of Trx1 antibody was validated by comparing the Trx1 expression in control and Trx1 knockdown cells (Fig. S2A–C). We observed that overexpressing TDP-43 dramatically decreases endogenous protein levels of Trx1 in N2a cells (Fig. 2A and B). We also observed that overexpressing hTDP-43 gradually decreases human Trx1 protein levels ectopically expressed in N2a cells in a dose-dependent manner (Fig. 2C and D).

**Fig. 2:**
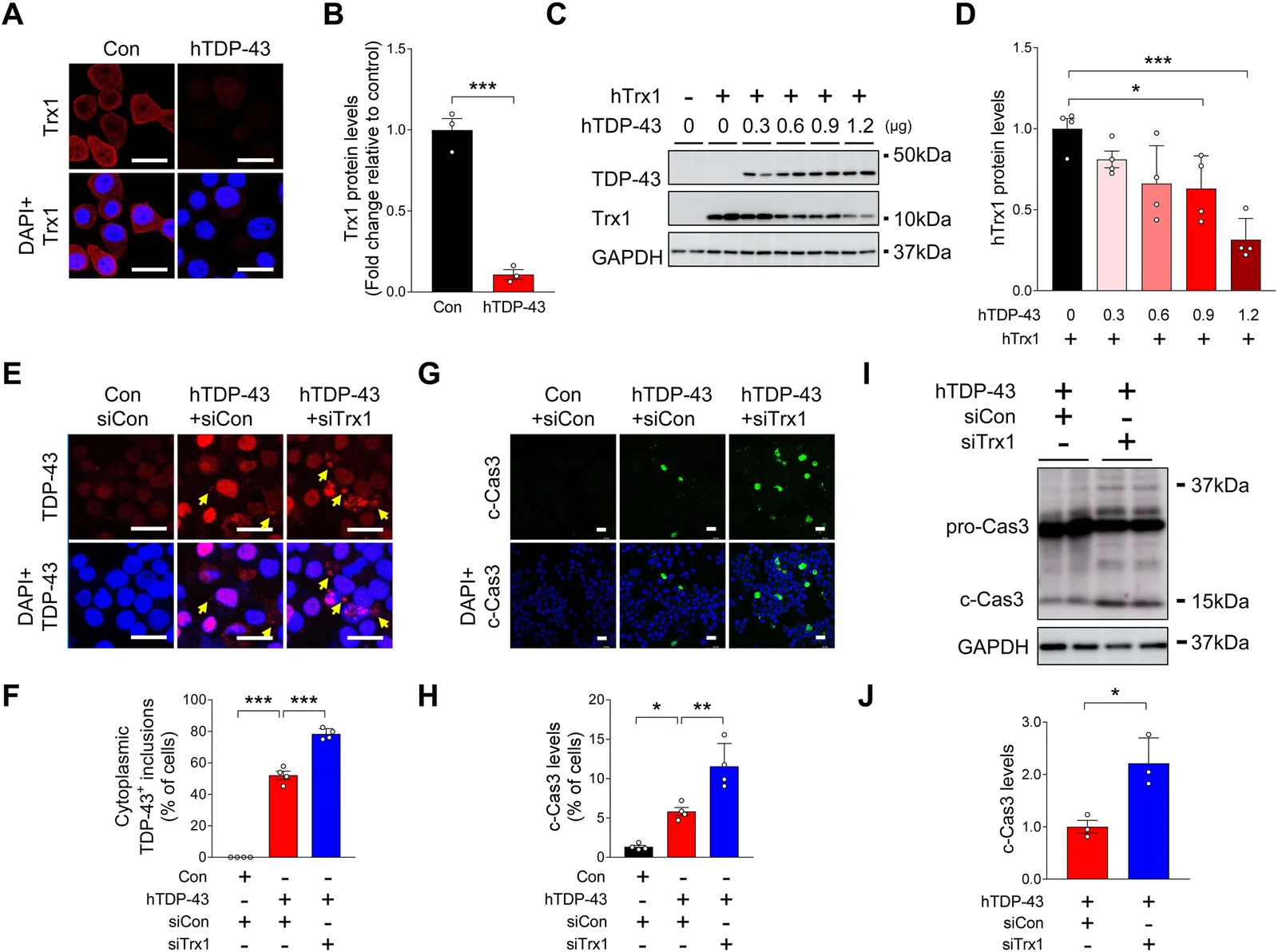
**Inhibition of Trx1 exacerbates TDP-43 pathology in N2a cells.** (A,B) Overexpressing TDP-43 decreases the levels of endogenous Trx1 protein in N2a cells. 48 h post-transfection, as indicated, endogenous Trx1 proteins were stained with *anti*-Trx1 antibody (A) and quantified (B). Each level was presented as a relative fold change to control (n = 3, unpaired *t-*test). (C,D) Overexpressing TDP-43 decreases the levels of Trx1 proteins ectopically expressed in N2a cells in a dose-dependent manner. 24 h post-plating, hTrx1 vectors were transfected to N2a cells with different amounts of hTDP-43 vectors as indicated. 48 h post-transfection, each protein level was analyzed by Western blot (C). Each Trx1 protein level was normalized to the corresponding GAPDH level and presented relative to the mean value of the control group (D) (n = 4, one-way ANOVA, *post-hoc* Tukey's test). (E,F) Inhibiting Trx1 significantly enhances cytoplasmic TDP-43 aggregation in N2a cells. 48 h post-transfection, N2a cells were stained with TDP-43-specific antibody (E). Yellow arrows indicate cytoplasmic TDP-43 inclusions. Cytoplasmic TDP-43 inclusions were normalized to the corresponding cell number and presented as a percentage of cells with cytoplasmic TDP-43 inclusions (F) (n = 4, one-way ANOVA, *post-hoc* Tukey's test). (G-J) Inhibiting Trx1 significantly enhances cleaved caspase 3 in N2a cells. 48 h post-transfection, cleaved caspase 3 (c-Cas3) was assessed by immunostaining with c-Cas3-specific antibody (G) and Western blot analysis with pan-caspase 3 antibody, which detects both pro-caspase-3 (pro-Cas3) and c-Cas3 (I). The percentages of c-Cas3-positive cells were quantified from immunostaining images (H), and c-Cas3 protein levels were quantified by Western blotting and normalized to GAPDH level and presented relative to the mean value of the control group (J) (H: n = 4, one-way ANOVA, *post-hoc* Tukey's test, J: n = 3, unpaired *t-*test). Nuclei were stained with DAPI (blue). All scale bars correspond to 25 μm. All values are means ± SEM. *p < 0.05, **p < 0.01, ***p < 0.001.

We next examined whether Trx1 indeed plays a role in TDP-43 pathology. We observed that inhibiting Trx1 significantly enhances cytoplasmic TDP-43 aggregation in N2a-hTDP-43 cells compared to the control (Fig. 2E and F). Trx1 inhibition also increases cleaved caspase 3 in N2a-hTDP-43 cells compared to the control, as demonstrated by immunostaining (Fig. 2G and H) and Western blot analysis (Fig. 2I and J). In contrast, overexpressing Trx1 strongly suppresses cytoplasmic TDP-43 aggregation (Fig. 3A and B). We further validated this finding by Western blot analysis. To do this, we analyzed the protein levels of soluble and insoluble TDP-43 by Western blot after Triton X-100 fractionation. Our results show that overexpressing Trx1 significantly decreases insoluble TDP-43 levels in N2a-hTDP-43 cells without altering soluble TDP-43 levels (Fig. S3A–C). Moreover, overexpressing Trx1 markedly decreases cleaved caspase 3 in N2a-hTDP-43 cells compared to the control (Fig. 3C–F). Taken together, our data indicate that Trx1 is protective against TDP-43 pathology in a neuronal cell model of TDP-43 overexpression.

**Fig. 3:**
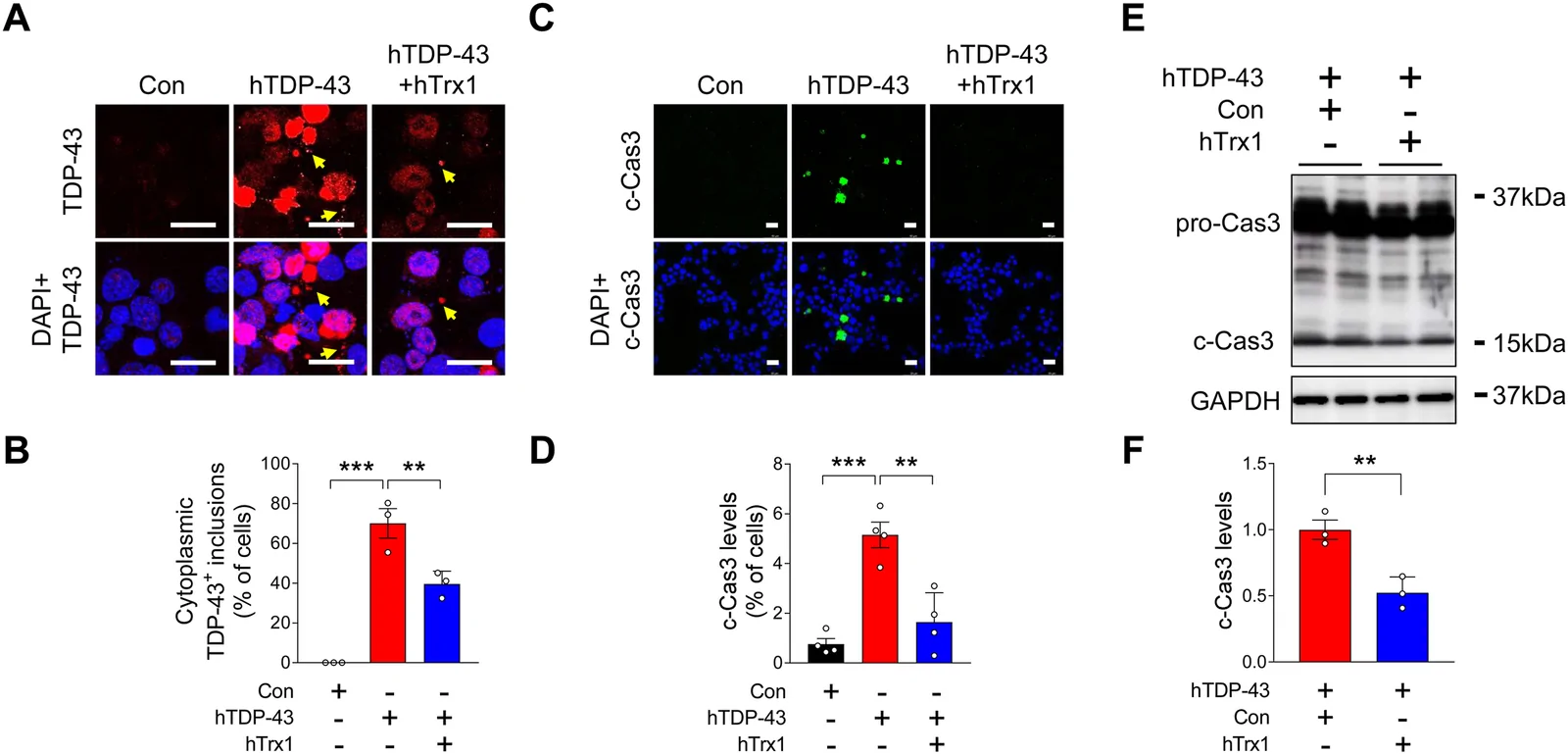
**Overexpression of Trx1 mitigates TDP-43 pathology in N2a cells.** (A,B) Overexpressing Trx1 significantly reduces cytoplasmic TDP-43 aggregation in N2a cells. 48 h post-transfection as indicated, N2a cells were stained with *anti*-TDP-43 (A). Yellow arrows indicate cytoplasmic TDP-43 inclusions. Cytoplasmic TDP-43 inclusions were normalized to the corresponding cell number and presented as a percentage of cells with cytoplasmic TDP-43 inclusions (B) (n = 3, one-way ANOVA, *post-hoc* Tukey's test). (C-F) Overexpressing Trx1 significantly reduces cleaved caspase 3 in N2a cells. 48 h post-transfection, c-Cas3 was assessed by immunostaining with c-Cas3-specific antibody (C) and Western blot analysis with pro-caspase-3 antibody (E). The percentages of c-Cas3-positive cells were quantified from immunostaining images (D), and c-Cas3 protein levels were quantified by Western blotting and normalized to the GAPDH level and presented relative to the mean value of the control group (F). (D: n = 4, one-way ANOVA, *post-hoc* Tukey's test, F: n = 3, unpaired *t-*test). Nuclei were stained with DAPI (blue). All scale bars correspond to 25 μm. All values are means ± SEM. *p < 0.05, **p < 0.01, ***p < 0.001.

### 3.3 Cytoplasmic TDP-43 sequesters *Trx1* mRNAs into insoluble TDP-43 inclusions, thereby preventing its translation

TDP-43 is a DNA/RNA binding protein that regulates target gene expression at multiple levels from transcription to translation. To investigate how TDP-43 overexpression suppresses Trx1 protein expression, we first examined whether overexpression of TDP-43 affects Trx1 at the transcriptional level. Our data showed that hTDP-43 overexpression does not affect the levels of endogenous *Trx1* mRNAs in N2a cells (Fig. S4A), suggesting that TDP-43 overexpression regulates Trx1 expression at the post-transcriptional level. We next examined the possibility that TDP-43 regulates Trx1 protein stability through direct interaction. However, we observed no physical interaction between TDP-43 and Trx1 at the protein level as demonstrated by immunoprecipitation (Fig. S4B and C) and proximity ligation assay (PLA) (Fig. S4D). HSP70, a well-established TDP-43-interacting protein [29], was used as a positive control in these assays. Several reports have shown that TDP-43 can bind to its target mRNAs, thereby regulating their translation [30], [31], [32], [33], [34]. Therefore, we next hypothesized that TDP-43 protein binds to *Trx1* mRNA and sequesters it into insoluble TDP-43 inclusions, thereby preventing its translation. To test this, we performed TDP-43 RNA-immunoprecipitation (RIP) using FLAG-specific antibody from N2a cells expressing hTDP-43 tagged with FLAG (N2a-hTDP-43-FLAG) and analyzed *Trx1* mRNA levels bound to TDP-43 proteins by qRT-PCR analysis (Fig. 4A–C). N2a cells expressing ectopic hTDP-43-5FL mutant, an established RNA-binding-deficient form of TDP-43 [22], [23], served as a negative control. The hTDP-43-5FL mutant carries five phenylalanine-to-leucine substitutions within the RNA recognition motifs (RRMs), thereby abolishing RNA binding. TDP-43 is known to regulate its expression through binding to its own mRNA [35]. In line with this, endogenous mouse *Tdp-*43 mRNAs bound to TDP-43 were enriched in N2a-hTDP-43-FLAG cells compared to the control (Fig. 4B), ensuring the validity of our TDP-43 RIP experiment. Interestingly, we found that endogenous *Trx1* mRNAs bound to hTDP-43-FLAG were enriched in N2a-hTDP-43-FLAG cells compared to the control (Fig. 4C), indicating the interaction of TDP-43 proteins with *Trx1* mRNAs. In contrast, TDP-43-5FL mutant proteins do not interact with endogenous mouse *Tdp-*43 mRNAs or *Trx1* mRNAs (Fig. 4B and C), supporting the conclusion that the RRMs of TDP-43 protein are required for the interaction between TDP-43 and *Trx1* mRNA. Consistent with a previous report [35], overexpression of human TDP-43 significantly reduced endogenous mouse TDP-43 protein levels, supporting the autoregulation of TDP-43 expression (Fig. 4D and E). Similarly, overexpression of human TDP-43 significantly reduced Trx1 protein levels, whereas the hTDP-43-5FL mutant has no significant effect on Trx1 expression (Fig. 4F and G).

**Fig. 4:**
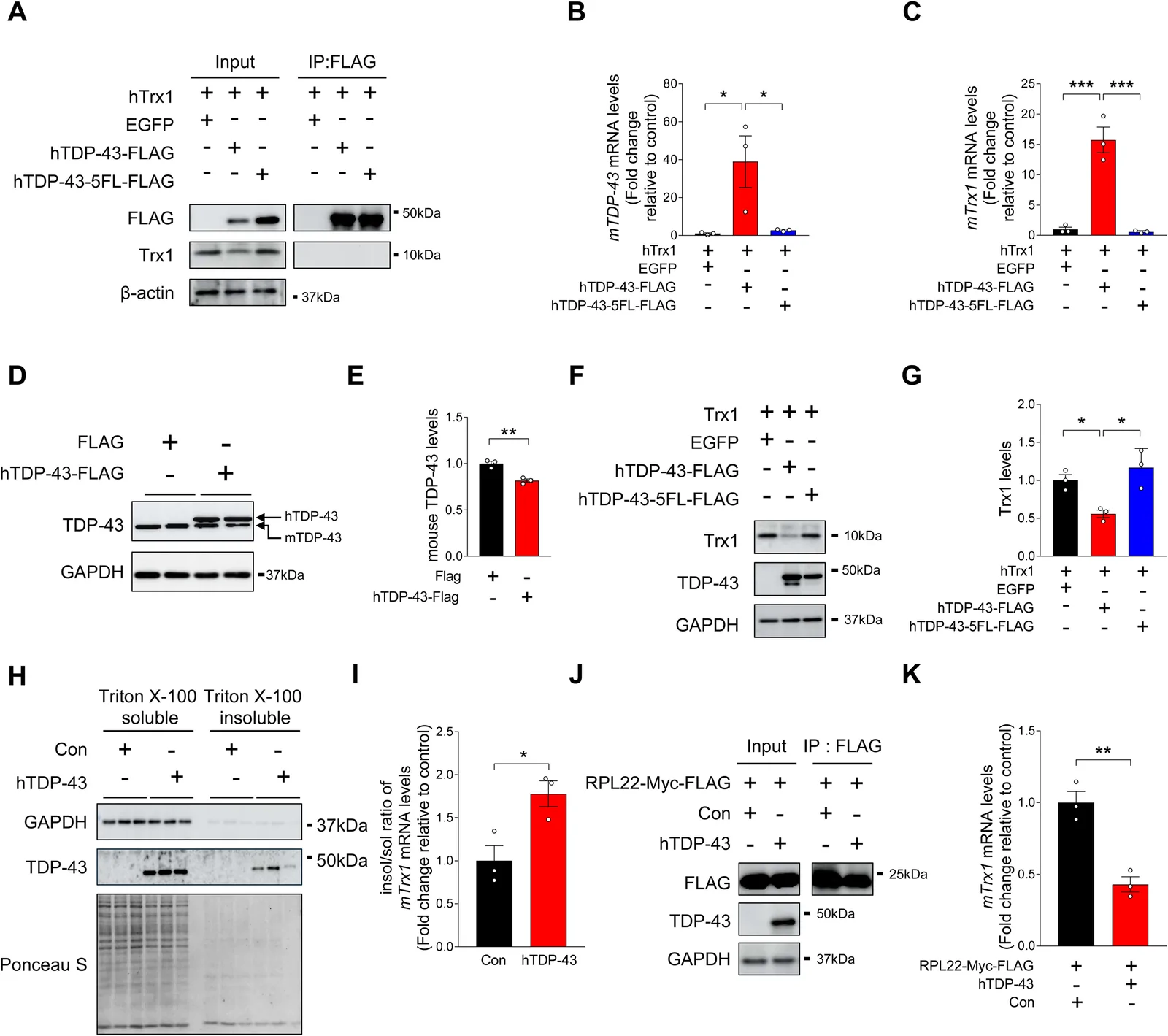
**Cytoplasmic TDP-43 sequesters *Trx1* mRNAs into insoluble TDP-43 inclusions, resulting in its translational suppression.** (A-C) TDP-43 protein binds to *Trx1* mRNA. For TDP-43 RIP, hTDP-43-FLAG proteins were immunoprecipitated using anti-FLAG antibody (A). N2a cells expressing ectopic hTDP-43-5FL mutant served as a negative control. Endogenous *Tdp-43* (B) and *Trx1* mRNAs (C) bound to TDP-43 proteins were analyzed by qRT-PCR. Each level was presented as a relative fold change compared to the control (n = 3, one-way ANOVA, *post-hoc* Tukey's test). (D,E) Overexpression of hTDP-43 induced autoregulation, resulting in reduced expression of endogenous mouse TDP-43.48 h post-transfection, TDP-43 was assessed by Western blot analysis with TDP-43-specific antibody which detects both human- and mouse TDP-43 (D). Endogenous mouse TDP-43 level was normalized by the corresponding GAPDH level and presented relative to the mean value of the control group (E) (n = 3, unpaired *t-*test). N2a cells expressing FLAG tag served as a negative control. (F,G) The RNA recognition motifs (RRMs) of TDP-43 are required for TDP-43-mediated regulation of Trx1 expression. 48 h post-transfection as indicated, Trx1 protein levels were analyzed by Western blot (F). Trx1 protein level was normalized to the corresponding GAPDH level and presented relative to the mean value of the control group (G) (n = 3, one-way ANOVA, *post-hoc* Tukey's test). (H,I) The insoluble-to-soluble ratio of *Trx1* mRNAs is significantly increased upon TDP-43 overexpression in N2a cells. 48 h post-transfection, cell lysates were first fractionated into nuclear and cytoplasmic fractions. The resulting cytoplasmic fractions were further fractioned into Triton X-100–soluble and –insoluble fractions as described in the Materials and Methods (H). The insoluble-to-soluble ratio of *Trx1* mRNAs is presented as a relative fold change to control (I) (n = 3, unpaired *t-*test). (J,K) The levels of *Trx1* mRNAs bound to ribosomes are significantly decreased upon TDP-43 overexpression in N2a cells. N2a cells were transiently transfected with FLAG-tagged RPL22 for ribosome isolation. 48 h post-transfection as indicated, ribosomes were immunoprecipitated using anti-FLAG antibody (J). N2a cells without hTDP-43 expression served as a control. The levels of *Trx1* mRNAs bound to ribosomes were measured by qRT-PCR and normalized by the corresponding levels of immunoprecipitated RPL22 protein. Each level was presented as a relative fold change to control (K) (n = 3, unpaired *t*-test). All values are means ± SEM. n.s. = non-significant, *p < 0.05, **p < 0.01, ***p < 0.001.

TDP-43 is known to regulate the nuclear export of its target mRNAs [4], [5]. Therefore, we examined whether overexpression TDP-43 affects the nuclear to cytoplasmic distribution of *Trx1* mRNAs. To do this, we separated the whole cell lysates into nuclear and cytoplasmic fractions and analyzed *Trx1* mRNA levels (Fig. S4E and F). We observed that the nuclear-to-cytoplasmic ratio of *Trx1* mRNAs is not altered by TDP-43 overexpression, suggesting that overexpressing TDP-43 does not affect the nuclear export of *Trx1* mRNAs in neuronal cells (Fig. S4F).

Cytoplasmic TDP-43 aggregates can sequester various target mRNAs into insoluble TDP-43 inclusions, thereby inhibiting their expression [30], [31], [32], [33], [34]. Therefore, we next examined whether *Trx1* mRNAs are enriched in the insoluble TDP-43 inclusions in N2a-hTDP-43 cells. To do this, we separated the cytoplasmic lysates into Triton X-100-soluble and -insoluble fractions and then analyzed *Trx1* mRNA levels (Fig. 4H and I). We observed that the insoluble-to-soluble ratio of *Trx1* mRNAs is significantly increased upon TDP-43 overexpression in N2a cells (Fig. 4I), indicating that *Trx1* mRNAs are sequestered into insoluble TDP-43 inclusions. Therefore, we hypothesized that sequestration of *Trx1* mRNAs into insoluble TDP-43 inclusions may result in the depletion of translationally active *Trx1* mRNAs in N2a-hTDP-43 cells. To test this, we pulled down ribosome-bound mRNAs by RPL22 RIP using FLAG-specific antibody from N2a cells expressing RPL22 tagged with FLAG (Fig. 4J). RPL22 incorporates into the 60S large ribosomal subunit and participates in mRNA translation [36]. We observed that the levels of ribosome-bound *Trx1* mRNAs are markedly decreased upon TDP-43 overexpression in N2a cells (Fig. 4K). Taken together, our results provide a novel mechanistic insight into the downregulation of Trx1 in TDP-43 proteinopathy, showing that cytoplasmic TDP-43 inhibits the translation of Trx1 by sequestering its mRNA into insoluble TDP-43 inclusions.

### 3.4 Suppression of Trx1 is a core mechanism underlying oxidative stress and mitochondrial dysfunction induced by TDP-43 overexpression

As aforementioned, oxidative stress plays a pivotal role in TDP-43 proteinopathy. Oxidative stress promotes TDP-43 pathology, including cytoplasmic TDP-43 aggregation and neurotoxicity. Meanwhile, TDP-43 proteinopathy itself disrupts redox homeostasis, thereby amplifying oxidative stress [20], [37], [38], [39], [40], [41]. Trx1 is a key redox-regulating enzyme that maintains cellular redox homeostasis [28]. Therefore, we examined whether Trx1 regulates oxidative stress in N2a-hTDP-43 cells. To do this, we measured ROS levels using redox-sensitive CellROX™ Deep Red dye (Fig. 5A–D). We observed that inhibiting Trx1 significantly enhances ROS levels in N2a-hTDP-43 cells compared to the control (Fig. 5A and B). In contrast, overexpressing Trx1 completely suppresses ROS induction by TDP-43 overexpression in N2a cells (Fig. 5C and D).

**Fig. 5:**
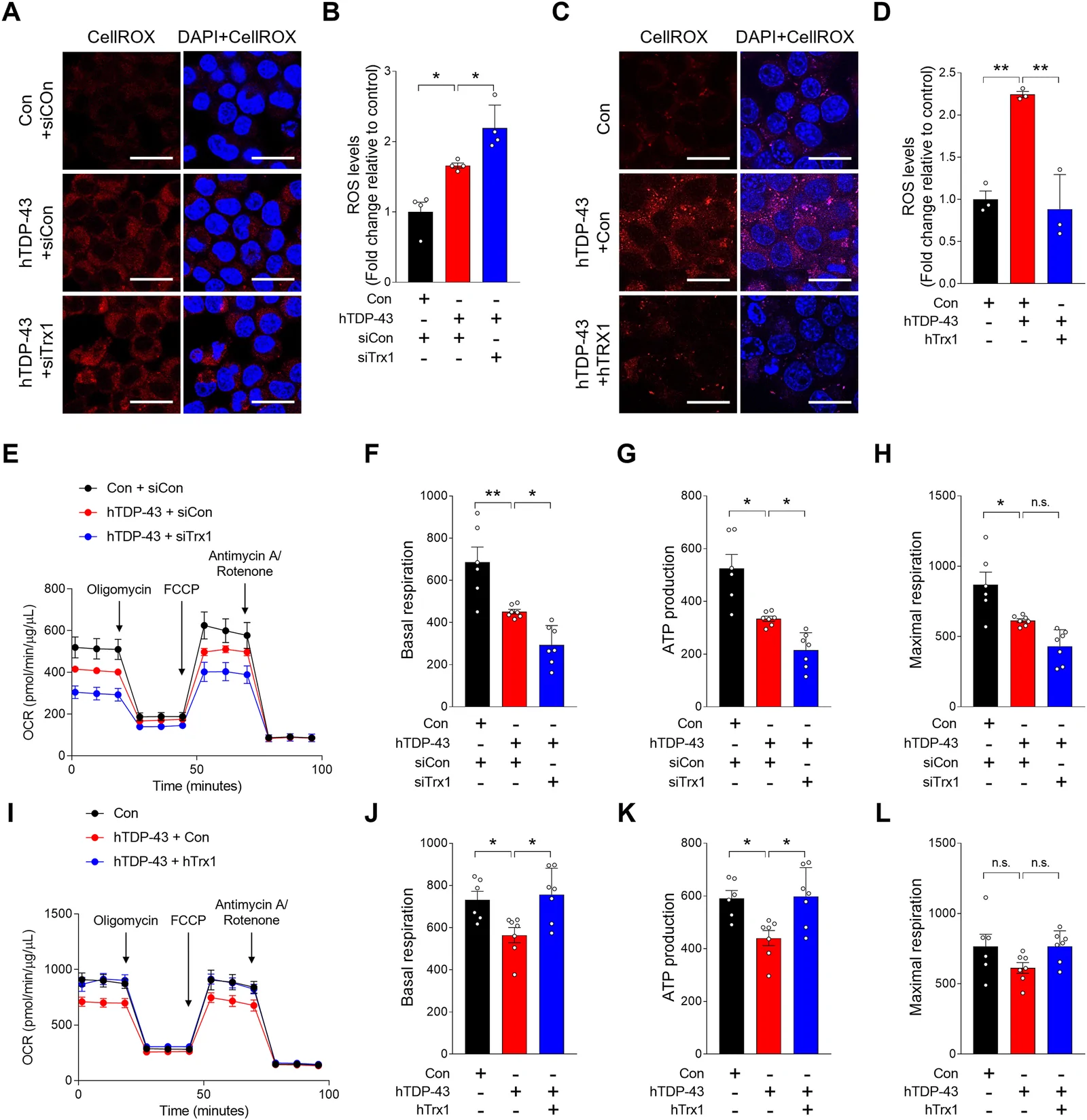
**Trx1 regulates oxidative stress and mitochondrial bioenergetics in a N2a model of TDP-43 overexpression.** (A,B) Inhibiting Trx1 significantly enhances ROS levels in N2a-hTDP-43 cells. 48 h post-transfection as indicated, N2a cells were stained with CellROX Deep Red dye for ROS (A). Nuclei were stained with DAPI (blue). Scale bars correspond to 25 μm. Each level was normalized to the corresponding cell number and presented as a relative fold change to control (B) (n = 4, one-way ANOVA, *post-hoc* Tukey's test). (C,D) Overexpressing Trx1 significantly reduces ROS levels in N2a-hTDP-43 cells. Each level was normalized to the corresponding cell number and presented as a relative fold change compared to control (D) (n = 3, one-way ANOVA, *post-hoc* Tukey's test). (E-H) Inhibiting Trx1 exacerbates mitochondrial dysfunction in N2a-hTDP-43 cells. The basal respiration (F), ATP production (G), and maximum respiratory capacity (H) were measured as described in the Materials and Methods (n = 6 - 7, one-way ANOVA, *post-hoc* Tukey's test). (I-L) Overexpressing Trx1 attenuates mitochondrial dysfunction in N2a-hTDP-43 cells (n = 6 ∼ 7, one-way ANOVA, *post-hoc* Tukey's test). All values are means ± SEM. n.s. = non-significant, *p < 0.05, **p < 0.01.

Mitochondria are a major source of ROS generation which occurs primarily through oxidative phosphorylation in the electron transport chain. Albeit some debates exist [42], we and others showed that aberrant accumulation of TDP-43 induces mitochondrial dysfunction [15], [20], [43]. Therefore, we examined whether Trx1 regulates mitochondrial bioenergetics in N2a-hTDP-43 cells. To do this, we measured the oxygen consumption rate using a Seahorse XF analyzer. In line with previous reports [20], TDP-43 overexpression significantly impairs mitochondrial bioenergetics, as evidenced by reduced basal respiration, ATP production, and maximum respiratory capacity in N2a cells (Fig. 5E–L). Importantly, inhibiting Trx1 exacerbates mitochondrial dysfunction in N2a-hTDP-43 cells compared to the control (Fig. 5E–H), whereas overexpressing Trx1 significantly attenuates mitochondrial defects (Fig. 5I–L). Taken together, our results suggest that suppression of Trx1 expression by cytoplasmic TDP-43 aggregates is a core molecular mechanism underlying oxidative stress and mitochondrial dysfunction induced by TDP-43 overexpression.

### 3.5 Overexpressing Trx1 attenuates TDP-43 pathology in primary neurons

Unlike immortalized N2a cells, neurons are highly polarized and energy-demanding cells that rely heavily on oxidative phosphorylation for energy generation, making them particularly susceptible to oxidative stress. For this reason, TDP-43 pathology and Trx1 function may differ between the two cell types, warranting validation of Trx1's protective effect in primary neurons. Therefore, we assessed the therapeutic potential of Trx1 overexpression against TDP-43 pathology in primary neurons. To establish a primary neuron model of TDP-43 overexpression, mouse primary cortical neurons were transduced with AAV expressing hTDP-43 (AAV-hTDP-43) at DIV7 and then analyzed the pathological changes at DIV14 (Fig. 6A). Primary neurons transduced with AAV control virus (AAV-Con) served as negative controls. As in N2a cells, we observed cytoplasmic TDP-43 aggregation in primary cortical neurons expressing hTDP-43 (Fig. S5A). We also assessed TDP-43-mediated neurotoxicity by analyzing total neurite outgrowth using an antibody specific for TUBB3, a neurite marker. In line with a previous report [44], primary neurons with ectopic TDP-43 expression exhibit neurite defects compared to the control (Fig. S5B and C). Using this model, we first examined whether TDP-43 overexpression suppresses Trx1 expression. As in N2a cells, TDP-43 overexpression significantly decreases endogenous Trx1 protein levels in primary cortical neurons (Fig. 6B and C). We next examined whether Trx1 overexpression suppresses TDP-43 aggregation. To do this, we analyzed the protein levels of soluble and insoluble TDP-43 by Western blot after Triton X-100 fractionation. Overexpression of Trx1 in primary neurons was achieved by transduction with AAV viruses expressing hTrx1 tagged with Myc-FLAG (AAV-hTRX1-Myc-FLAG) (Fig. S5D). Our results show that overexpressing Trx1 substantially diminishes insoluble TDP-43 levels in primary neurons expressing hTDP-43 without altering soluble TDP-43 levels (Fig. 6D–F), suggesting that Trx1 inhibits TDP-43 aggregation. In addition, overexpressing Trx1 markedly decreases ROS levels induced by TDP-43 overexpression in primary neurons (Fig. 6G and H). Importantly, overexpressing Trx1 completely rescues the neurite defect in primary neurons transduced with AAV-hTDP-43 (Fig. 6I and J). Taken together, our results demonstrated that inducing Trx1 expression strongly attenuates TDP-43 pathology in primary neurons overexpressing hTDP-43.

**Fig. 6:**
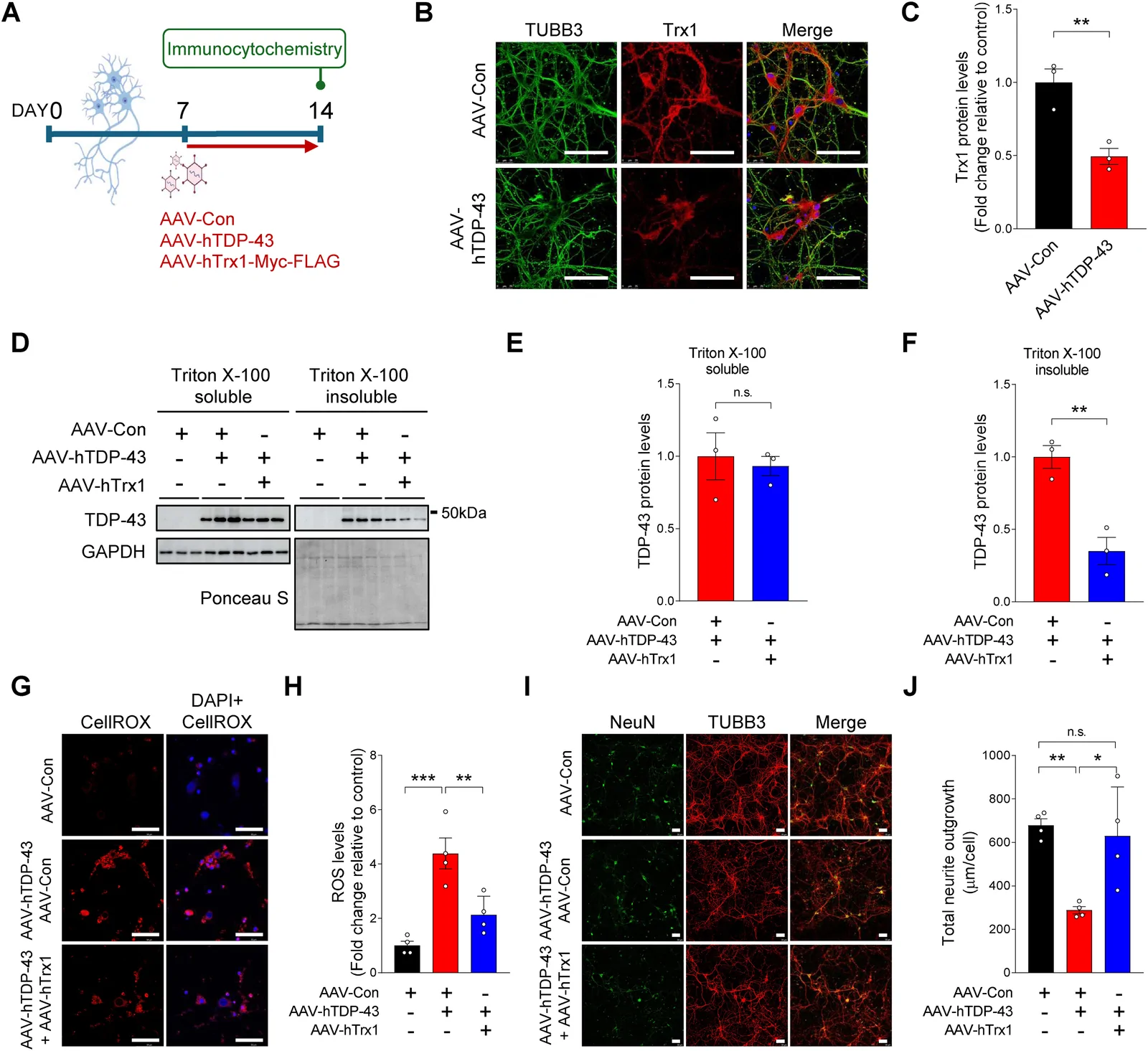
**Overexpression of Trx1 attenuates hTDP-43-mediated neurotoxicity in primary cortical neurons. (**A) Schematic diagram of virus transduction in primary cortical neurons. (B,C) Overexpressing TDP-43 decreases the levels of endogenous Trx1 protein in mouse primary cortical neurons. AAV-hTDP-43 were transduced to mouse primary cortical neurons at DIV7. Neurons were stained with *anti*-Trx1 and anti-MAP2 antibodies (B). Scale bars correspond to 50 μm. AAV control virus (AAV-Con) served as negative control. Each Trx1 level was normalized to the corresponding cell number and presented as a relative fold change compared to control (C) (n = 3, unpaired *t-*test). (D-F) Overexpressing Trx1 decreases insoluble TDP-43 levels in primary cortical neurons. AAV-hTDP-43 viruses were transduced to mouse primary cortical neurons with/without AAV-hTrx1-Myc-FLAG viruses at DIV7. TDP-43 protein levels were assessed by Western blot in Triton X-100-soluble (E) and -insoluble fractions (F) (n = 3, unpaired *t-*test). Soluble- and insoluble TDP-43 levels were normalized to the corresponding GAPDH and Ponceau S levels, respectively. Data are presented relative to the mean value of the group transduced solely with AAV-hTDP-43 (n = 3, unpaired *t-*test). (G,H) Overexpressing Trx1 significantly decreases ROS levels in primary cortical neurons. Neurons were stained with CellROX Deep Red dye (red) (G). Total intensities of ROS were normalized to the corresponding cell number (H) (n = 4, one-way ANOVA, *post-hoc* Tukey's test). (I,J) Overexpressing Trx1 rescues TDP-43-associated neurite outgrowth defect in primary cortical neurons. Neurons were stained with TUBB3 (red), a neurite marker (I). Total neurite outgrowth length was quantified using MetaMorph analysis and normalized to the corresponding cell number (J) (n = 4, one-way ANOVA, post-hoc Tukey's test). Nuclei were stained with DAPI (blue). Scale bars correspond to 50 μm. All values are means ± SEM. n.s. = non-significant. *p < 0.05, **p < 0.01, ***p < 0.001.

### 3.6 Overexpressing Trx1 attenuates oxidative stress and TDP-43 aggregation in human iPSC-derived motor neurons harboring pathogenic TDP-43 M337V mutation

Although TDP-43 overexpression models have provided important mechanistic insights, they are limited by excessive TDP-43 levels, which may produce non-physiological or artifactual phenotypes. Moreover, non-human models may not adequately reflect human-specific molecular regulation and cellular vulnerability associated with TDP-43 pathology. In contrast, iPSC-MNs provide a more physiologically relevant condition with endogenous TDP-43 expression and human genetic context. Previously, we reported the successful generation of human iPSC-MN harboring the ALS-associated TDP-43 M337V mutation (TDP-43^M337V^ iPSC-MN) from an isogenic control iPSC line with TDP-43 WT by genetic editing [45]. We validated the differentiation of iPSC into MNs by staining the cells with *anti*-ISL-1/2 and -HB9 antibodies, motor-neuronal markers, along with *anti*-TUBB3 antibody, a differentiated neuronal marker (Fig. 7A and B). In line with previous studies [11], [45], [46], we observed that TDP-43^M337V^ iPSC-MNs develop more cytoplasmic TDP-43 inclusions compared to the isogenic TDP-43^WT^ control (Fig. 7C and D). Moreover, ROS levels significantly increased in TDP-43^M337V^ iPSC-MNs compared to TDP-43^WT^ iPSC-MNs (Fig. 7E and F). Consistent with this, Trx1 protein levels were drastically decreased in TDP-43^M337V^ iPSC-MNs compared to the isogenic control (Fig. 7G and H).

**Fig. 7:**
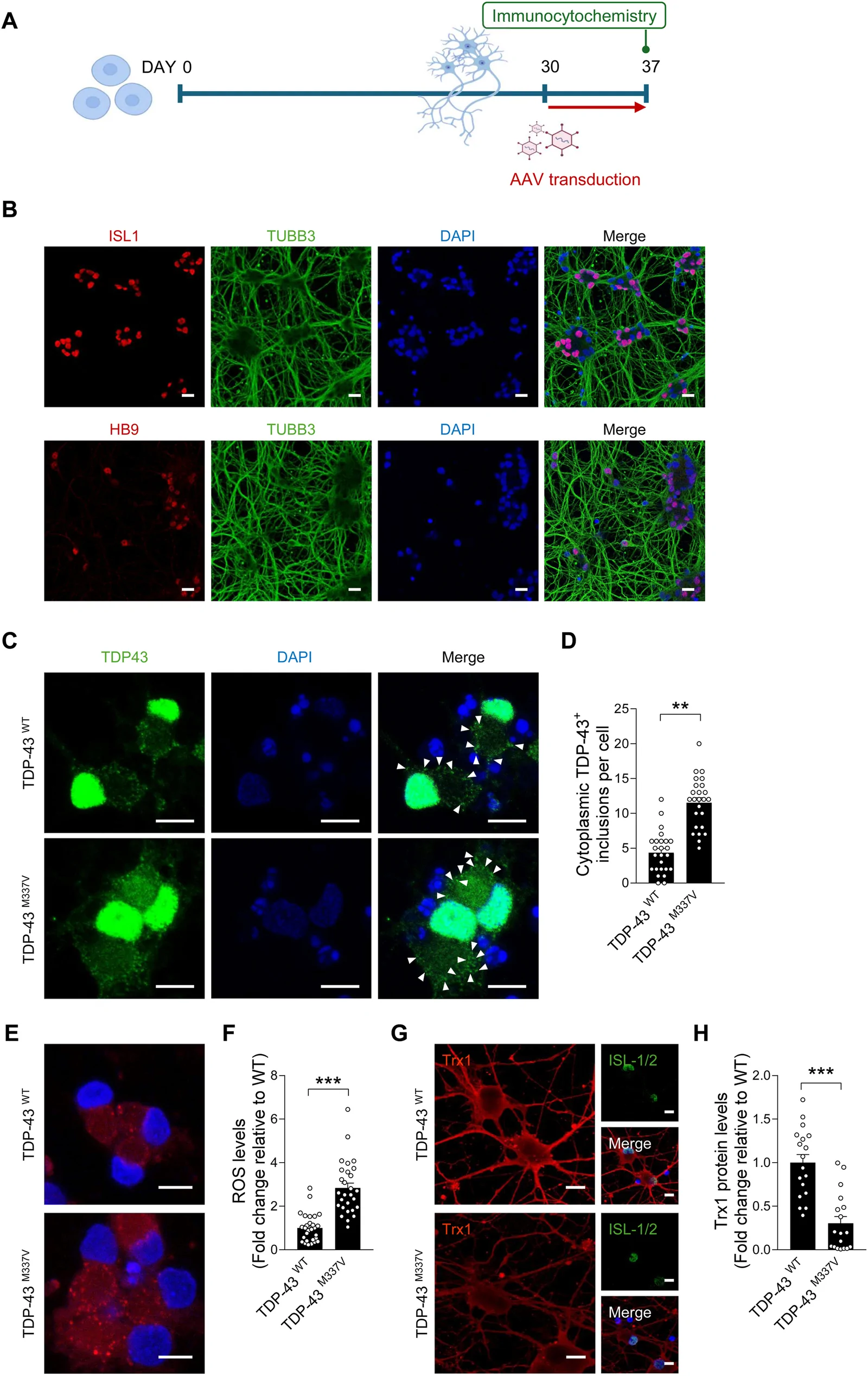
**Oxidative stress is elevated, and Trx1 is reduced in TDP-43^M337V^ iPSC-MNs compared to isogenic controls.** (A) Schematic diagram of iPSC-MN differentiation and virus transduction. (B) Representative immunofluorescent image of iPSC-MNs after 30 days of differentiation stained with ISL-1/2 (upper panel, red), HB9 (lower panel, red), TUBB3 (both, green), and DAPI (blue). Scale bars correspond to 20 μm. (C,D) TDP-43^M337V^ iPSC-MNs develop more cytoplasmic TDP-43 inclusions compared to the isogenic TDP-43^WT^ control. iPSC-MNs were treated with 0.4 M sorbitol for 1.5 h and stained with *anti*-TDP-43 antibody (green) and DAPI (blue) on day 37 of differentiation (C). Arrow heads indicate cytoplasmic TDP-43 inclusions. TDP-43 inclusions were normalized to the corresponding cell numbers (D) (n = 25, unpaired *t*-test). (E,F) ROS levels are significantly increased in TDP-43^M337V^ iPSC-MNs compared to the isogenic TDP-43^WT^ control. Neurons were stained with CellROX Deep Red dye (red) (E). Total intensities of ROS were normalized to the corresponding cell numbers (F) and presented as relative fold changes compared to the TDP-43^WT^ control (n = 28, unpaired *t*-test). (G,H) Trx1 protein levels are significantly decreased in TDP-43^M337V^ iPSC-MNs compared to isogenic TDP-43^WT^ controls. Neurons were stained with ISL-1/2 (green), Trx1 (red), and DAPI (blue) (G). Total intensities of endogenous Trx1 protein were normalized to the corresponding cell numbers and presented as relative fold changes compared to the TDP-43^WT^ control (H) (n = 18, unpaired *t*-test). All values are means ± SEM. **p < 0.01, ***p < 0.001. Scale bars correspond to 10 μm (C,E,G).

We next assessed whether inducing Trx1 prevents cytoplasmic TDP-43 aggregation in TDP-43^M337V^ iPSC-MNs. To do this, we transduced iPSC-MNs with AAV-hTrx1-Myc-FLAG on day 30 of differentiation and verified a successful induction of Trx1 expression on day 37 of differentiation (Fig. 8A). Overexpressing Trx1 strongly attenuates cytoplasmic TDP-43 aggregation in TDP-43^M337V^ iPSC-MNs (Fig. 8A and B). Furthermore, we observed that Trx1 overexpression dramatically decreases ROS levels in TDP-43^M337V^ iPSC-MNs (Fig. 8C and D). Our findings suggest that the pathological role and the therapeutic potential of Trx1 in TDP-43 proteinopathy are conserved in the human genetic context.

**Fig. 8:**
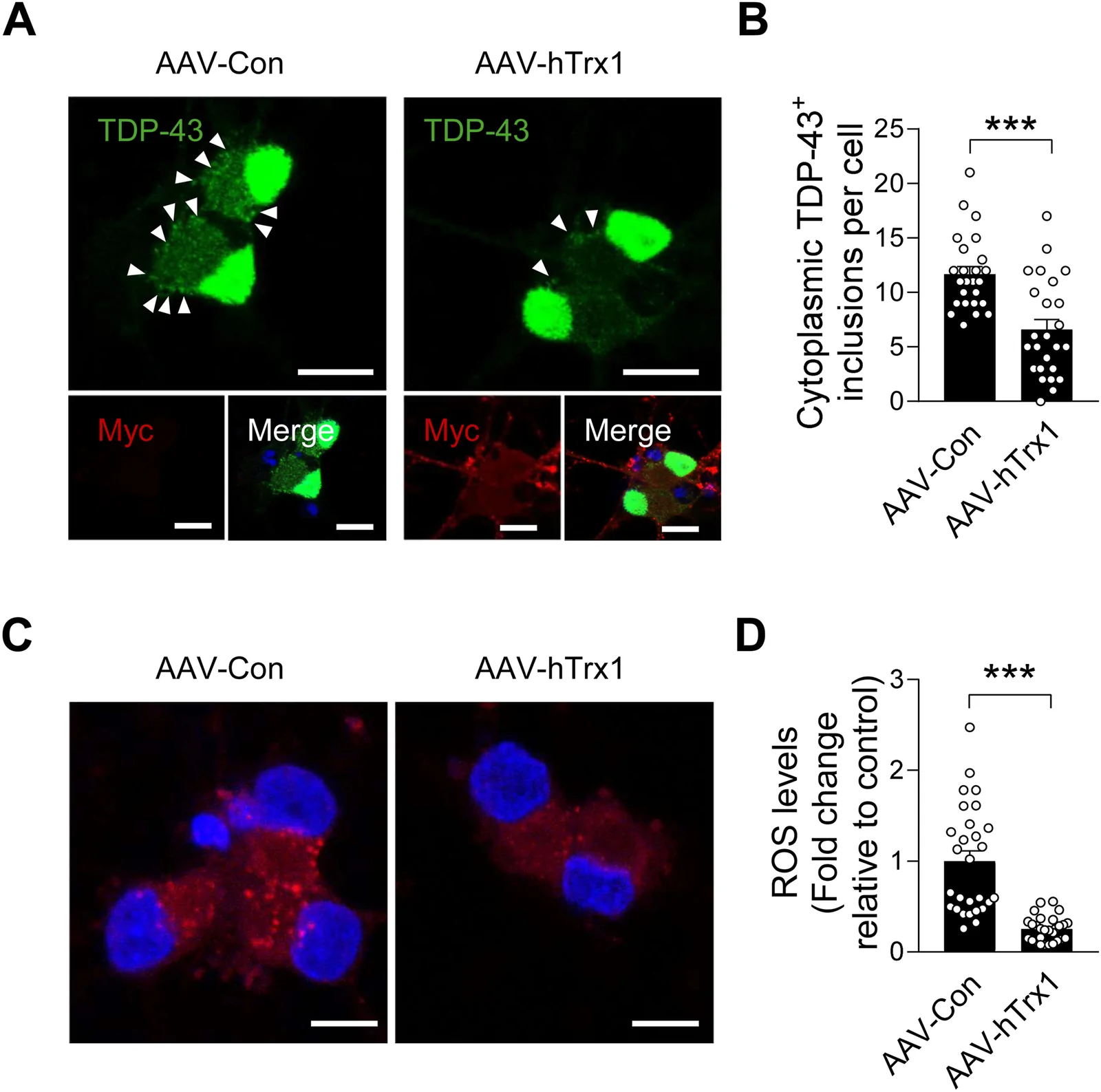
**Overexpression of Trx1 suppresses cytoplasmic TDP-43 aggregation and oxidative stress in TDP-43^M337V^ mutant iPSC-MNs.** (A,B) Overexpressing Trx1 markedly reduces cytoplasmic TDP-43 aggregation in TDP-43^M337V^ iPSC-MNs. AAV-hTrx1-Myc-FLAG or AAV-Con viruses were transduced to TDP-43^M337V^ iPSC-MNs on day 30 of differentiation. iPSC-MNs were treated with 0.4 M sorbitol for 1.5 h and stained with *anti*-TDP-43 (green), *anti*-Myc antibody (red), and DAPI (blue) on day 37 of differentiation (A). Arrow heads indicate cytoplasmic TDP-43 inclusions. TDP-43 inclusions were normalized to the corresponding cell numbers (B) (n = 25, unpaired *t*-test). (C,D) Overexpressing Trx1 dramatically reduces ROS levels in TDP-43^M337V^ iPSC-MNs. Neurons were stained with CellROX Deep Red dye (red) (C). Total intensities of ROS were normalized to the corresponding cell numbers and presented as relative fold changes compared to control (D) (n = 28, unpaired *t*-test). Scale bars correspond to 10 μm. All values are means ± SEM. ***p < 0.001.

## 4 Discussion

A common pathological hallmark of TDP-43 proteinopathies is progressive neuronal loss accompanied by nuclear depletion, cytoplasmic mislocalization, and aggregation of TDP-43. While loss of nuclear TDP-43 function and the resulting disruption of RNA metabolism are widely recognized as central mechanisms underlying ALS and other TDP-43 proteinopathies, accumulating evidence also supports a complementary contribution of cytoplasmic TDP-43 aggregation through toxic gain-of-function mechanisms. However, the molecular pathways linking cytoplasmic TDP-43 aggregation to neuronal dysfunction and degeneration remain incompletely understood. Because cytoplasmic accumulation of TDP-43 represents a major pathological hallmark of ALS and other TDP-43 proteinopathies, experimental models that reproducibly induce cytoplasmic TDP-43 aggregation provide a useful approach for investigating the downstream molecular consequences of this pathology. In this study, we performed comprehensive proteomic profiling to elucidate the molecular characteristics and core mechanisms underlying TDP-43-associated neuropathology using an N2a model of hTDP-43 overexpression. Our model was designed to isolate the contribution of cytoplasmic TDP-43 aggregation to downstream pathological processes. Although this overexpression-based model does not fully recapitulate all aspects of TDP-43 proteinopathy, particularly nuclear depletion of TDP-43, it reproduces several key pathological features associated with cytoplasmic TDP-43 aggregation, including oxidative stress, mitochondrial dysfunction, and neurotoxicity. We observed no detectable stress granule formation in our model, indicating that cytoplasmic TDP-43 aggregation occurs in the absence of stress granule formation in this experimental context. This observation is consistent with previous studies showing that, although stress granules can facilitate the local concentration of TDP-43 and, under certain conditions, promote its phase transition during prolonged or severe cellular stress, they are not an absolute prerequisite for TDP-43 aggregation. Indeed, multiple studies have demonstrated that cytoplasmic TDP-43 can undergo mislocalization, phase separation, and pathological aggregation independently of stress granule assembly [37], [47], [48]. Thus, this model provides a useful experimental platform for investigating the molecular mechanisms underlying cytoplasmic TDP-43-mediated neurotoxicity. Of note, proteomic analysis showed that our model recapitulates key molecular alterations of TDP-43 proteinopathy, including dysregulations of RNA metabolism, proteostasis, mitochondrial function, and redox homeostasis, as previously reported [2], [4], [6], [27]. Focusing on the underlying molecular mechanisms of TDP-43-associated neurotoxicity, we identified Trx1 as a key regulator of mitochondrial bioenergetics and redox homeostasis via integrated analyses of the dysregulated proteome. Using primary mouse neurons, we further demonstrated that Trx1 protects against TDP-43 pathology. Importantly, the reduction of Trx1 expression was also reproduced in human iPSC-MN models which express endogenous levels of TDP-43. Moreover, Trx1 induction significantly attenuates cytoplasmic TDP-43 aggregation and oxidative stress in human iPSC-MN models, suggesting that our findings are not solely attributable to the overexpression-based nature of the N2a and primary neuron model. Instead, these findings support the involvement of Trx1 dysregulation in TDP-43 proteinopathy as a shared mechanism across different experimental models. Translational suppression of Trx1 by cytoplasmic TDP-43 aggregation contributes to increased oxidative stress and mitochondrial dysfunction, ultimately leading to neurotoxicity (Fig. 9).

**Fig. 9:**
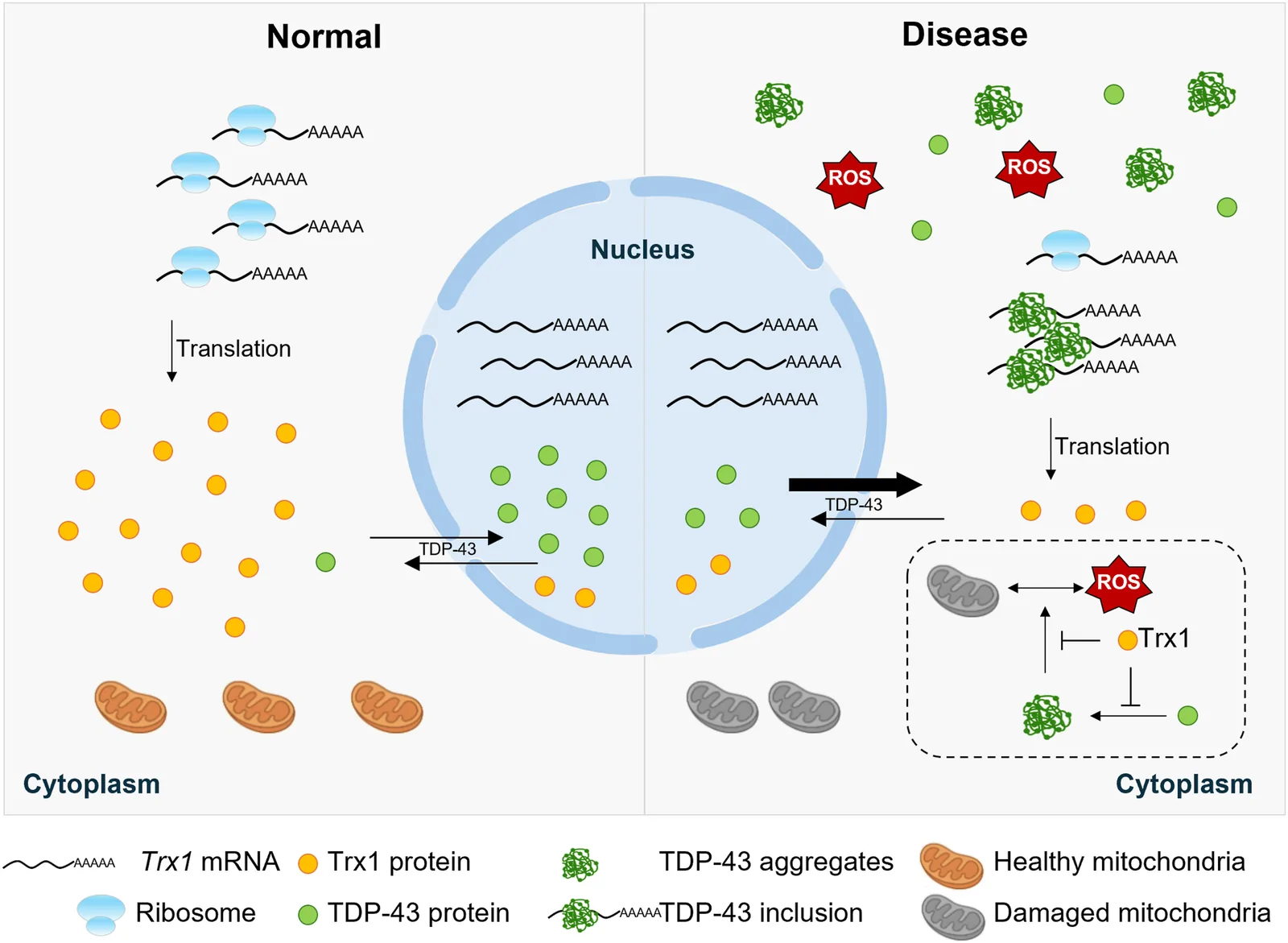
**A proposed role of Trx1 in TDP-43 proteinopathy.** Under disease conditions, TDP-43 undergoes cytoplasmic mislocalization and aggregation, leading to oxidative stress, mitochondrial dysfunction, and ultimately neurotoxicity in neurons. In TDP-43 proteinopathy, cytoplasmic TDP-43 elicits oxidative stress and mitochondrial dysfunction by sequestering *Trx1* mRNAs within insoluble TDP-43 inclusions, thereby suppressing its translation. Conversely, Trx1 protects against TDP-43 proteinopathy by reducing TDP-43 aggregation and oxidative stress, thereby mitigating neurotoxicity.

As aforementioned, oxidative stress plays a critical role in TDP-43 proteinopathy. Trx1 is a key redox-regulating enzyme that maintains cellular redox homeostasis by reducing oxidized proteins and scavenging reactive oxygen species [28]. Therefore, it is critical to understand the regulatory mechanisms of Trx1 expression and maintain the levels of functional Trx1 especially in neurons, which are vulnerable to oxidative stress [49], [50]. Importantly, we identified a novel dysregulation mechanism of Trx1 by cytoplasmic TDP-43 aggregation that cytoplasmic TDP-43 protein sequesters *Trx1* mRNA into insoluble TDP-43 inclusions through direct interaction, ultimately leading to its translational suppression. Given the pivotal role of Trx1 in redox homeostasis, our results indicate that cytoplasmic TDP-43 aggregation elicits oxidative stress by direct inhibition of Trx1 expression, which in turn promotes further aggregation of TDP-43, thus creating a vicious feed-forward loop. Supporting this idea, we observed that inducing Trx1 expression substantially reduces oxidative stress and cytosolic TDP-43 aggregation, leading to neuroprotection in TDP-43 proteinopathy. Notably, our results further showed that Trx1 overexpression predominantly reduces insoluble TDP-43 without substantially affecting soluble TDP-43 levels. This finding raises the possibility that the protective effects of Trx1 may extend beyond its role in alleviating oxidative stress and may involve a more direct modulation of TDP-43 aggregation dynamics. Specifically, Trx1 may preferentially limit the formation, maturation, or persistence of insoluble TDP-43 aggregates, while having relatively little effect on the stability, turnover, or steady-state levels of soluble TDP-43. Thus, Trx1 may ameliorate TDP-43 proteinopathy not only by restoring redox homeostasis but also by modulating the aggregation state of TDP-43 itself. Although the precise molecular mechanisms underlying this potential aggregation-related function of Trx1 remain to be elucidated, these findings suggest that Trx1 may act at multiple levels to disrupt the pathogenic interplay between oxidative stress and TDP-43 aggregation. Trx1 dysregulation represents a key mechanism linking TDP-43 aggregation to redox imbalance in our models. However, our findings do not exclude the possibility that cytoplasmic TDP-43 aggregation also contributes to oxidative stress through additional mechanisms independent of Trx1 dysregulation. Future studies investigating these complementary pathways will further clarify the complex interplay between TDP-43 aggregation and oxidative stress in TDP-43 proteinopathy. Under physiological conditions, nuclear TDP-43 binds to its target pre-mRNAs and mature mRNAs, regulating their splicing and stability. In addition, cytoplasmic TDP-43 is known to regulate axonal transport and local translation of target mRNAs in neurons [51]. In the context of diseases, TDP-43 undergoes mislocalization and cytoplasmic aggregation, leading to both loss-of-function and toxic gain-of-function effects. For instance, axonal transport of target mRNAs mediated by cytoplasmic TDP-43 is impaired by ALS-causing mutations in motor neurons [52], suggesting loss-of-function effect. In contrast, similar to Trx1, cytoplasmic TDP-43 aggregates have been demonstrated to sequester various target mRNAs into insoluble TDP-43 inclusions, thereby inhibiting their translation [30], [31], [32], [33], [34], supporting toxic gain-of-function effects. Thus, cytoplasmic TDP-43 aggregates are not just epiphenomena but active mediators of cellular toxicity. Our findings provide a novel mechanistic insight into the toxic gain-of-function effects of cytoplasmic TDP-43 aggregation.

It is of note that mitochondrial dysfunction and oxidative stress are early events before the onset of severe pathology and behavioral deficits in animal models of TDP-43 proteinopathy [53], [54], [55]. Accumulating evidence suggests that oxidative stress may function both as an upstream driver and a downstream effector of TDP-43 aggregation [3], [17], [18], [56]. Oxidative insults can initiate TDP-43 misfolding, cytoplasmic mislocalization, and aggregation, while TDP-43 pathology itself disrupts mitochondrial integrity, redox homeostasis, and protein quality control, thereby amplifying oxidative burden [20], [37], [38], [39], [40], [41]. Mitochondria generate bioenergy through oxidative phosphorylation, which produces ROS as a byproduct. It is well known that mitochondrial dysfunction amplifies oxidative stress, and vice versa. Aberrant accumulation of TDP-43 within mitochondria could induce mitochondrial dysfunction, ultimately increasing oxidative stress [15], [43]. This bidirectional interplay creates a vicious feed-forward loop that drives neuronal degeneration, yet the precise temporal sequence and molecular pathways remain unresolved. Although our findings indicate that Trx1 effectively restores mitochondrial function, it is yet unclear whether this restorative effect of Trx1 arises predominantly from the amelioration of oxidative stress, the reduction of cytoplasmic TDP-43 aggregation, and/or alternative mechanistic routes. Given that TDP-43 has been reported to perturb mitochondrial homeostasis through diverse mechanisms, including sequestration of regulatory miRNAs, transcriptional dysregulation of mitochondrial genes, and aberrant interactions with mitochondrial proteins [20], [37], [38], [39], [40], [41], further comprehensive investigations are warranted to delineate the mechanisms linking Trx1 to mitochondrial integrity and oxidative stress in TDP-43 proteinopathy.

The pathogenic role of Trx1 in primary TDP-43 proteinopathies, such as ALS and FTD, remains unexplored. Notably, studies with post-mortem tissues and preclinical models have shown that Trx1 is dysregulated and neuroprotective in various secondary TDP-43 proteinopathies, such as AD, PD, HD, and TBI [57], [58], [59], [60], [61], [62], [63], [64], [65]. However, due to the complex pathological processes beyond TDP-43 pathology, it remains unclear whether there is a direct link between TDP-43 pathology and Trx1 dysregulation in those secondary TDP-43 proteinopathies. In this study, we provide novel mechanistic insight into the pathological link between Trx1 and TDP-43 proteinopathy. We identified a novel toxic gain-of-function mechanism whereby TDP-43 aggregation leads to translational suppression of Trx1, resulting in cytoplasmic TDP-43 aggregates leads to increased oxidative stress, mitochondrial dysfunction, and ultimately neurotoxicity. It is noteworthy that oxidative stress is commonly associated with the pathogeneses of both primary- and secondary TDP-43 proteinopathies [14], [15]. Given the broad association of TDP-43 aggregation with various neurodegenerative diseases, our findings highlight that dysregulation of Trx1 by cytoplasmic TDP-43 aggregates might be a converging driving force of TDP-43-associated pathogenesis as a key node of redox imbalance.

## CRediT authorship contribution statement

**Ji Min Lee:** Writing – review & editing, Writing – original draft, Validation, Methodology, Investigation, Formal analysis, Data curation. **Eugene Bok:** Writing – review & editing, Writing – original draft, Validation, Methodology, Investigation, Formal analysis, Data curation. **Hyeonjin Jeon:** Writing – review & editing, Writing – original draft, Validation, Methodology, Investigation, Formal analysis, Data curation. **Seonhyeok Hwang:** Writing – review & editing, Writing – original draft, Validation, Methodology, Investigation, Formal analysis, Data curation. **Jiwon Hong:** Writing – review & editing, Writing – original draft, Validation, Methodology, Investigation, Formal analysis, Data curation. **Sung Bae Lee:** Writing – review & editing, Writing – original draft, Methodology, Investigation, Funding acquisition, Conceptualization. **Sun Joo Cha:** Validation, Formal analysis, Data curation. **Soo Hyun Kang:** Validation, Formal analysis, Data curation, Funding acquisition. **Hyung-Jun Kim:** Writing – review & editing, Methodology, Funding acquisition. **Mookyung Cheon:** Writing – review & editing, Methodology, Funding acquisition. **Byung-Hoon Lee:** Writing – review & editing, Methodology, Funding acquisition. **Chuna Kim:** Writing – review & editing, Writing – original draft, Validation, Supervision, Resources, Project administration, Funding acquisition. **Sang-Won Lee:** Writing – review & editing, Writing – original draft, Validation, Supervision, Resources, Project administration, Funding acquisition. **Sang Ryong Kim:** Writing – review & editing, Writing – original draft, Validation, Supervision, Resources, Project administration, Funding acquisition. **Minyeop Nahm:** Writing – review & editing, Writing – original draft, Validation, Supervision, Resources, Project administration, Funding acquisition, Conceptualization. **Jaekwang Kim:** Writing – review & editing, Writing – original draft, Validation, Supervision, Resources, Project administration, Funding acquisition, Conceptualization.

## Funding

This work was supported by 10.13039/100018749KBRI basic research program through 10.13039/100018749Korea Brain Research Institute funded by 10.13039/501100014188Ministry of Science and ICT (25-BR-02-03 to H-J.K., M.C., M.N., J.K., 25-BR-02-05 to H-J.K., M.C., J.K., 25-BR-02-09 to S·H·K., 25-BR-04-01 to M.N., J.K.), 10.13039/501100003725National Research Foundation of Korea (NRF) grant funded by the Korea Government (10.13039/501100014188MSIT) (2021R1F1A1049849, RS-2026-25492655 to J.K., RS-2025-16065773 to S.R.K., RS-2026-25524860 to J.K., S.R.K, RS-2025-16652968 to S.-W.L., RS-2024-00409972 to S·B.L., B·H.L.), Korea 10.13039/100018696Health Technology R&D Project through the 10.13039/501100003710Korea Health Industry Development Institute (10.13039/501100003710KHIDI) and 10.13039/100020206Korea Dementia Research Center (KDRC) funded by the 10.13039/100009647Ministry of Health & Welfare and 10.13039/501100014188Ministry of Science and ICT (HU21C0027 to S·B.L., B-H.L., J.K.), and 10.13039/501100008783National Research Council of Science & Technology (10.13039/501100008783NST) Aging Convergence Research Center and the 10.13039/501100003715KRIBB Research Initiative Program (CRC22015−300, KGM1132612 to C·K.)

## Declaration of competing interest

The authors declare that they have no known competing financial interests or personal relationships that could have appeared to influence the work reported in this paper.

## Data Availability

Data will be made available on request.

## References

[1] Ou S.H., Wu F., Harrich D., Garcia-Martinez L.F., Gaynor R.B. (1995). Cloning and characterization of a novel cellular protein, TDP-43, that binds to human immunodeficiency virus type 1 TAR DNA sequence motifs. J. Virol..

[2] Suk T.R., Rousseaux M.W.C. (2020). The role of TDP-43 mislocalization in amyotrophic lateral sclerosis. Mol. Neurodegener..

[3] Balendra R., Sreedharan J., Hallegger M., Luisier R., Lashuel H.A., Gregory J.M., Patani R. (2025). Amyotrophic lateral sclerosis caused by TARDBP mutations: from genetics to TDP-43 proteinopathy. Lancet Neurol..

[4] Klim J.R., Pintacuda G., Nash L.A., Guerra San Juan I., Eggan K. (2021). Connecting TDP-43 pathology with neuropathy. Trends Neurosci..

[5] de Boer E.M.J., Orie V.K., Williams T., Baker M.R., De Oliveira H.M., Polvikoski T., Silsby M., Menon P., van den Bos M., Halliday G.M., van den Berg L.H., Van Den Bosch L., van Damme P., Kiernan M.C., van Es M.A., Vucic S. (2020). TDP-43 proteinopathies: a new wave of neurodegenerative diseases. J. Neurol. Neurosurg. Psychiatry.

[6] Prasad A., Bharathi V., Sivalingam V., Girdhar A., Patel B.K. (2019). Molecular mechanisms of TDP-43 misfolding and pathology in amyotrophic lateral sclerosis. Front. Mol. Neurosci..

[7] Keating S.S., San Gil R., Swanson M.E.V., Scotter E.L., Walker A.K. (2022). TDP-43 pathology: from noxious assembly to therapeutic removal. Prog. Neurobiol..

[8] Ding Q., Chaplin J., Morris M.J., Hilliard M.A., Wolvetang E., Ng D.C.H., Noakes P.G. (2021). TDP-43 mutation affects stress granule dynamics in differentiated NSC-34 motoneuron-like cells. Front. Cell Dev. Biol..

[9] Huang C., Yan S., Zhang Z. (2020). Maintaining the balance of TDP-43, mitochondria, and autophagy: a promising therapeutic strategy for neurodegenerative diseases. Transl. Neurodegener..

[10] Lee E.B., Lee V.M., Trojanowski J.Q. (2011). Gains or losses: molecular mechanisms of TDP43-mediated neurodegeneration. Nat. Rev. Neurosci..

[11] Bilican B., Serio A., Barmada S.J., Nishimura A.L., Sullivan G.J., Carrasco M., Phatnani H.P., Puddifoot C.A., Story D., Fletcher J., Park I.H., Friedman B.A., Daley G.Q., Wyllie D.J., Hardingham G.E., Wilmut I., Finkbeiner S., Maniatis T., Shaw C.E., Chandran S. (2012). Mutant induced pluripotent stem cell lines recapitulate aspects of TDP-43 proteinopathies and reveal cell-specific vulnerability. Proc. Natl. Acad. Sci. U. S. A..

[12] Smith A.S.T., Chun C., Hesson J., Mathieu J., Valdmanis P.N., Mack D.L., Choi B.O., Kim D.H., Bothwell M. (2021). Human induced pluripotent stem cell-derived TDP-43 mutant neurons exhibit consistent functional phenotypes across multiple gene edited lines despite transcriptomic and splicing discrepancies. Front. Cell Dev. Biol..

[13] Lee S., Jeon Y.M., Cha S.J., Kim S., Kwon Y., Jo M., Jang Y.N., Lee S., Kim J., Kim S.R., Lee K.J., Lee S.B., Kim K., Kim H.J. (2020). PTK2/FAK regulates UPS impairment via SQSTM1/p62 phosphorylation in TARDBP/TDP-43 proteinopathies. Autophagy.

[14] Barnham K.J., Masters C.L., Bush A.I. (2004). Neurodegenerative diseases and oxidative stress. Nat. Rev. Drug Discov..

[15] Cunha-Oliveira T., Montezinho L., Mendes C., Firuzi O., Saso L., Oliveira P.J., Silva F.S.G. (2020). Oxidative stress in amyotrophic lateral sclerosis: pathophysiology and opportunities for pharmacological intervention. Oxid. Med. Cell. Longev..

[16] Ayala V., Granado-Serrano A.B., Cacabelos D., Naudi A., Ilieva E.V., Boada J., Caraballo-Miralles V., Llado J., Ferrer I., Pamplona R., Portero-Otin M. (2011). Cell stress induces TDP-43 pathological changes associated with ERK1/2 dysfunction: implications in ALS. Acta Neuropathol..

[17] Iguchi Y., Katsuno M., Takagi S., Ishigaki S., Niwa J., Hasegawa M., Tanaka F., Sobue G. (2012). Oxidative stress induced by glutathione depletion reproduces pathological modifications of TDP-43 linked to TDP-43 proteinopathies. Neurobiol. Dis..

[18] Ratti A., Gumina V., Lenzi P., Bossolasco P., Fulceri F., Volpe C., Bardelli D., Pregnolato F., Maraschi A., Fornai F., Silani V., Colombrita C. (2020). Chronic stress induces formation of stress granules and pathological TDP-43 aggregates in human ALS fibroblasts and iPSC-motoneurons. Neurobiol. Dis..

[19] Moujalled D., Grubman A., Acevedo K., Yang S., Ke Y.D., Moujalled D.M., Duncan C., Caragounis A., Perera N.D., Turner B.J., Prudencio M., Petrucelli L., Blair I., Ittner L.M., Crouch P.J., Liddell J.R., White A.R. (2017). TDP-43 mutations causing amyotrophic lateral sclerosis are associated with altered expression of RNA-Binding protein hnRNP K and affect the Nrf2 antioxidant pathway. Hum. Mol. Genet..

[20] Jeon Y.M., Kwon Y., Lee S., Kim S., Jo M., Lee S., Kim S.R., Kim K., Kim H.J. (2021). Vitamin B12 reduces TDP-43 toxicity by alleviating oxidative stress and mitochondrial dysfunction. Antioxidants.

[21] Mikuriya S., Takegawa-Araki T., Tamura M. (2025). Edaravone mitigates TDP-43 mislocalization in human amyotrophic lateral sclerosis neurons with potential implication of the SIRT1-XBP1 pathway. Free Radic. Biol. Med..

[22] Mann J.R., Gleixner A.M., Mauna J.C., Gomes E., DeChellis-Marks M.R., Needham P.G., Copley K.E., Hurtle B., Portz B., Pyles N.J., Guo L., Calder C.B., Wills Z.P., Pandey U.B., Kofler J.K., Brodsky J.L., Thathiah A., Shorter J., Donnelly C.J. (2019). RNA binding antagonizes neurotoxic phase transitions of TDP-43. Neuron.

[23] Buratti E., Baralle F.E. (2001). Characterization and functional implications of the RNA binding properties of nuclear factor TDP-43, a novel splicing regulator of CFTR exon 9. J. Biol. Chem..

[24] Kang C., Huh S., Nam D., Kim H., Hong J., Hwang D., Lee S.W. (2022). Novel online three-dimensional separation expands the detectable functional landscape of cellular phosphoproteome. Anal. Chem..

[25] Madar I.H., Ko S.I., Kim H., Mun D.G., Kim S., Smith R.D., Lee S.W. (2017). Multiplexed post-experimental monoisotopic mass refinement (mPE-MMR) to increase sensitivity and accuracy in peptide identifications from Tandem Mass Spectra of cofragmentation. Anal. Chem..

[26] Hyeon D.Y., Nam D., Han Y., Kim D.K., Kim G., Kim D., Bae J., Back S., Mun D.G., Madar I.H., Lee H., Kim S.J., Kim H., Hyun S., Kim C.R., Choi S.A., Kim Y.R., Jeong J., Jeon S., Choo Y.W., Lee K.B., Kwon W., Choi S., Goo T., Park T., Suh Y.A., Kim H., Ku J.L., Kim M.S., Paek E., Park D., Jung K., Baek S.H., Jang J.Y., Hwang D., Lee S.W. (2023). Proteogenomic landscape of human pancreatic ductal adenocarcinoma in an Asian population reveals tumor cell-enriched and immune-rich subtypes. Nat. Cancer.

[27] Wu H.Y., Wang L.C., Sow B.M., Leow D., Zhu J., Gallo K.M., Wilsbach K., Gupta R., Ostrow L.W., Yeo C.J.J., Sobota R.M., Li R. (2024). TDP43 aggregation at ER-exit sites impairs ER-to-Golgi transport. Nat. Commun..

[28] Lu J., Holmgren A. (2014). The thioredoxin antioxidant system. Free Radic. Biol. Med..

[29] Gu J., Wang C., Hu R., Li Y., Zhang S., Sun Y., Wang Q., Li D., Fang Y., Liu C. (2021). Hsp70 chaperones TDP-43 in dynamic, liquid-like phase and prevents it from amyloid aggregation. Cell Res..

[30] Bjork R.T., Mortimore N.P., Loganathan S., Zarnescu D.C. (2022). Dysregulation of translation in TDP-43 proteinopathies: deficits in the RNA supply chain and local protein production. Front. Neurosci..

[31] Coyne A.N., Lorenzini I., Chou C.C., Torvund M., Rogers R.S., Starr A., Zaepfel B.L., Levy J., Johannesmeyer J., Schwartz J.C., Nishimune H., Zinsmaier K., Rossoll W., Sattler R., Zarnescu D.C. (2017). Post-transcriptional inhibition of Hsc70-4/HSPA8 expression leads to synaptic vesicle cycling defects in multiple models of ALS. Cell Rep..

[32] Altman T., Ionescu A., Ibraheem A., Priesmann D., Gradus-Pery T., Farberov L., Alexandra G., Shelestovich N., Dafinca R., Shomron N., Rage F., Talbot K., Ward M.E., Dori A., Kruger M., Perlson E. (2021). Axonal TDP-43 condensates drive neuromuscular junction disruption through inhibition of local synthesis of nuclear encoded mitochondrial proteins. Nat. Commun..

[33] Sephton C.F., Cenik C., Kucukural A., Dammer E.B., Cenik B., Han Y., Dewey C.M., Roth F.P., Herz J., Peng J., Moore M.J., Yu G. (2011). Identification of neuronal RNA targets of TDP-43-containing ribonucleoprotein complexes. J. Biol. Chem..

[34] Colombrita C., Onesto E., Megiorni F., Pizzuti A., Baralle F.E., Buratti E., Silani V., Ratti A. (2012). TDP-43 and FUS RNA-binding proteins bind distinct sets of cytoplasmic messenger RNAs and differently regulate their post-transcriptional fate in motoneuron-like cells. J. Biol. Chem..

[35] Ayala Y.M., De Conti L., Avendaño-Vázquez S.E., Dhir A., Romano M., D'Ambrogio A., Tollervey J., Ule J., Baralle M., Buratti E., Baralle F.E. (2011). TDP-43 regulates its mRNA levels through a negative feedback loop. EMBO J..

[36] Sanz E., Yang L., Su T., Morris D.R., McKnight G.S., Amieux P.S. (2009). Cell-type-specific isolation of ribosome-associated mRNA from complex tissues. Proc. Natl. Acad. Sci. U. S. A..

[37] Zuo X., Zhou J., Li Y., Wu K., Chen Z., Luo Z., Zhang X., Liang Y., Esteban M.A., Zhou Y., Fu X.D. (2021). TDP-43 aggregation induced by oxidative stress causes global mitochondrial imbalance in ALS. Nat. Struct. Mol. Biol..

[38] Yu C.H., Davidson S., Harapas C.R., Hilton J.B., Mlodzianoski M.J., Laohamonthonkul P., Louis C., Low R.R.J., Moecking J., De Nardo D., Balka K.R., Calleja D.J., Moghaddas F., Ni E., McLean C.A., Samson A.L., Tyebji S., Tonkin C.J., Bye C.R., Turner B.J., Pepin G., Gantier M.P., Rogers K.L., McArthur K., Crouch P.J., Masters S.L. (2020). TDP-43 triggers mitochondrial DNA release via mPTP to activate cGAS/STING in ALS. Cell.

[39] Xu Y.F., Gendron T.F., Zhang Y.J., Lin W.L., D'Alton S., Sheng H., Casey M.C., Tong J., Knight J., Yu X., Rademakers R., Boylan K., Hutton M., McGowan E., Dickson D.W., Lewis J., Petrucelli L. (2010). Wild-type human TDP-43 expression causes TDP-43 phosphorylation, mitochondrial aggregation, motor deficits, and early mortality in transgenic mice. J. Neurosci..

[40] Wang P., Deng J., Dong J., Liu J., Bigio E.H., Mesulam M., Wang T., Sun L., Wang L., Lee A.Y., McGee W.A., Chen X., Fushimi K., Zhu L., Wu J.Y. (2019). TDP-43 induces mitochondrial damage and activates the mitochondrial unfolded protein response. PLoS Genet..

[41] Gao J., Wang L., Yan T., Perry G., Wang X. (2019). TDP-43 proteinopathy and mitochondrial abnormalities in neurodegeneration. Mol. Cell. Neurosci..

[42] Kawamata H., Peixoto P., Konrad C., Palomo G., Bredvik K., Gerges M., Valsecchi F., Petrucelli L., Ravits J.M., Starkov A., Manfredi G. (2017). Mutant TDP-43 does not impair mitochondrial bioenergetics in vitro and in vivo. Mol. Neurodegener..

[43] Dafinca R., Barbagallo P., Talbot K. (2021). The role of mitochondrial dysfunction and ER stress in TDP-43 and C9ORF72 ALS. Front. Cell. Neurosci..

[44] Herzog J.J., Deshpande M., Shapiro L., Rodal A.A., Paradis S. (2017). TDP-43 misexpression causes defects in dendritic growth. Sci. Rep..

[45] Park N.Y., Heo Y., Yang J.W., Yoo J.M., Jang H.J., Jo J.H., Park S.J., Lin Y., Choi J., Jeon H., Cha S.J., Bae G., Kim D., Kim J., Zeno W., Park J.B., Isozumi N., Saio T., Kim S.H., Lee H., Hong B.H., Nahm M., Lee Y.H., Hong Y.B. (2025). Graphene quantum dots attenuate TDP-43 proteinopathy in amyotrophic lateral sclerosis. ACS Nano.

[46] Egawa N., Kitaoka S., Tsukita K., Naitoh M., Takahashi K., Yamamoto T., Adachi F., Kondo T., Okita K., Asaka I., Aoi T., Watanabe A., Yamada Y., Morizane A., Takahashi J., Ayaki T., Ito H., Yoshikawa K., Yamawaki S., Suzuki S., Watanabe D., Hioki H., Kaneko T., Makioka K., Okamoto K., Takuma H., Tamaoka A., Hasegawa K., Nonaka T., Hasegawa M., Kawata A., Yoshida M., Nakahata T., Takahashi R., Marchetto M.C., Gage F.H., Yamanaka S., Inoue H. (2012). Drug screening for ALS using patient-specific induced pluripotent stem cells. Sci. Transl. Med..

[47] Fernandes N., Nero L., Lyons S.M., Ivanov P., Mittelmeier T.M., Bolger T.A., Buchan J.R. (2020). Stress granule assembly can facilitate but is not required for TDP-43 cytoplasmic aggregation. Biomolecules.

[48] Yan X., Kuster D., Mohanty P., Nijssen J., Pombo-Garcia K., Garcia Morato J., Rizuan A., Franzmann T.M., Sergeeva A., Ly A.M., Liu F., Passos P.M., George L., Wang S.H., Shenoy J., Danielson H.L., Ozguney B., Honigmann A., Ayala Y.M., Fawzi N.L., Dickson D.W., Rossoll W., Mittal J., Alberti S., Hyman A.A. (2025). Intra-condensate demixing of TDP-43 inside stress granules generates pathological aggregates. Cell.

[49] Silva-Adaya D., Gonsebatt M.E., Guevara J. (2014). Thioredoxin system regulation in the central nervous system: experimental models and clinical evidence. Oxid. Med. Cell. Longev..

[50] Ren X., Zou L., Zhang X., Branco V., Wang J., Carvalho C., Holmgren A., Lu J. (2017). Redox signaling mediated by thioredoxin and glutathione systems in the central nervous System. Antioxidants Redox Signal..

[51] Nagano S., Jinno J., Abdelhamid R.F., Jin Y.S., Shibata M., Watanabe S., Hirokawa S., Nishizawa M., Sakimura K., Onodera O., Okada H., Okada T., Saito Y., Takahashi-Fujigasaki J., Murayama S., Wakatsuki S., Mochizuki H., Araki T. (2020). TDP-43 transports ribosomal protein mRNA to regulate axonal local translation in neuronal axons. Acta Neuropathol..

[52] Alami N.H., Smith R.B., Carrasco M.A., Williams L.A., Winborn C.S., Han S.S.W., Kiskinis E., Winborn B., Freibaum B.D., Kanagaraj A., Clare A.J., Badders N.M., Bilican B., Chaum E., Chandran S., Shaw C.E., Eggan K.C., Maniatis T., Taylor J.P. (2014). Axonal transport of TDP-43 mRNA granules is impaired by ALS-Causing mutations. Neuron.

[53] Wang W., Li L., Lin W.L., Dickson D.W., Petrucelli L., Zhang T., Wang X. (2013). The ALS disease-associated mutant TDP-43 impairs mitochondrial dynamics and function in motor neurons. Hum. Mol. Genet..

[54] Stribl C., Samara A., Trumbach D., Peis R., Neumann M., Fuchs H., Gailus-Durner V., Hrabe de Angelis M., Rathkolb B., Wolf E., Beckers J., Horsch M., Neff F., Kremmer E., Koob S., Reichert A.S., Hans W., Rozman J., Klingenspor M., Aichler M., Walch A.K., Becker L., Klopstock T., Glasl L., Holter S.M., Wurst W., Floss T. (2014). Mitochondrial dysfunction and decrease in body weight of a transgenic knock-in mouse model for TDP-43. J. Biol. Chem..

[55] Gautam M., Gunay A., Chandel N.S., Ozdinler P.H. (2022). Mitochondrial dysregulation occurs early in ALS motor cortex with TDP-43 pathology and suggests maintaining NAD(+) balance as a therapeutic strategy. Sci. Rep..

[56] Onda-Ohto A., Hasegawa-Ogawa M., Matsuno H., Shiraishi T., Bono K., Hiraki H., Kanegae Y., Iguchi Y., Okano H.J. (2023). Specific vulnerability of iPSC-derived motor neurons with TDP-43 gene mutation to oxidative stress. Mol. Brain.

[57] Arodin L., Lamparter H., Karlsson H., Nennesmo I., Bjornstedt M., Schroder J., Fernandes A.P. (2014). Alteration of thioredoxin and glutaredoxin in the progression of Alzheimer's disease. J. Alzheimers Dis..

[58] Lovell M.A., Xie C., Gabbita S.P., Markesbery W.R. (2000). Decreased thioredoxin and increased thioredoxin reductase levels in Alzheimer's disease brain. Free Radic. Biol. Med..

[59] Awan M.U.N., Yan F., Mahmood F., Bai L., Liu J., Bai J. (2022). The Functions of Thioredoxin 1 in Neurodegeneration. Antioxidants Redox Signal..

[60] Bjorklund G.R., Wong J.N.F., Brafman D., Bowser R., Stabenfeldt S.E. (2023). Traumatic brain injury induces TDP-43 mislocalization and neurodegenerative effects in tissue distal to the primary injury site in a non-transgenic mouse. Acta Neuropathol. Commun..

[61] Jia J., Sheng Z., Zhang Y., Guo L., Chen Z., Zhu D., Zeng X., Liu H. (2025). Thioredoxin-1 inhibits NLRP3-mediated pyroptosis by regulating TXNIP in models of Alzheimer's disease. Sci. Rep..

[62] Liu Z., Ye Q., Wang F., Guo Y., Cui R., Wang J., Wang D. (2021). Overexpression of thioredoxin reductase 1 can reduce DNA damage, mitochondrial autophagy and endoplasmic reticulum stress in Parkinson's disease. Exp. Brain Res..

[63] Gu R., Bai L., Yan F., Zhang S., Zhang X., Deng R., Zeng X., Sun B., Hu X., Li Y., Bai J. (2024). Thioredoxin-1 decreases alpha-synuclein induced by MPTP through promoting autophagy-lysosome pathway. Cell Death Discov..

[64] Fox J., Lu Z., Barrows L. (2015). Thiol-disulfide oxidoreductases TRX1 and TMX3 decrease neuronal atrophy in a lentiviral mouse model of Huntington's Disease. PLoS Curr.

[65] Chen C. (2024). TDP-43 is a key molecule accelerating development of Alzheimer's disease following traumatic brain injury. Neural Regen. Res..

