# Supplementary material for: Cytoplasmic TDP-43 aggregation promotes oxidative stress and neurotoxicity through Trx1 mRNA sequestration and translational suppression
Source: Redox Biol. 2026 Nov 4;98:104469. doi: 10.1016/j.redox.2026.104469 (PMC13769236; doi:10.1016/j.redox.2026.104469)
Supplement: Supplementary file 1 [file mmc1.docx]

**Supplementary Information**

**Cytoplasmic TDP-43 aggregation promotes oxidative stress and neurotoxicity through Trx1 mRNA sequestration and translational suppression**

Ji Min Lee^a,b,1^, Eugene Bok^a,1^, Hyeonjin Jeon^a,1^, Seonhyeok Hwang^c,1^, Jiwon Hong^d,1^, Sung Bae Lee^e,1^, Sun Joo Cha^a^, Soo Hyun Kang^a^, Hyung-Jun Kim^a^, Mookyung Cheon^a^, Byung-Hoon Lee^f^, Chuna Kim^c,^*, Sang-Won Lee^d,g,h^**, Sang Ryong Kim^b,i^***, Minyeop Nahm^a,^****, Jaekwang Kim^a,^*****

^a^Dementia Research Group, Korea Brain Research Institute, Daegu 41068, Republic of Korea

^b^School of Life Sciences and Biotechnology, BK21 FOUR KNU Creative BioResearch Group, Kyungpook National University, Daegu 41566, Republic of Korea.

^c^Aging Convergence Research Center, Korea Research Institute of Bioscience and Biotechnology, Daejeon 34141, Republic of Korea.

^d^Department of Chemistry, Center for Proteogenome Research, Korea University, Seoul 02841, Republic of Korea.

^e^Department of Brain Sciences, DGIST, Daegu 42988, Republic of Korea.

^f^Department of New Biology, DGIST, Daegu 42988, Republic of Korea.

^g^TargetX Inc., Seoul 02841, Republic of Korea.

^h^National Research Laboratory for Convergence Degradation Biology (NRLDB), Seoul 02841, Republic of Korea.

^i^Brain Science and Engineering Institute, Kyungpook National University, Daegu 41944, Korea

*Corresponding authors. Aging Convergence Research Center, Korea Research Institute of Bioscience and Biotechnology, 125, Gwahak-ro, Yuseong-gu, Daejeon 34141, Republic of Korea 5Department of Bioinformatics, KRIBB School of Bioscience, Korea University of Science and Technology (UST), 217, Gajeong-ro, Yuseong-gu, Daejeon 34113, Republic of Korea

**Corresponding authors. Department of Chemistry and Center for Proteogenome Research, Korea University, 145, Anam-ro, Seongbuk-gu, Seoul 02841, Republic of Korea.

***Corresponding authors. School of Life Science and Biotechnology, BK21 FOUR KNU Creative BioResearch Group, Brain Science and Engineering Institute, Kyungpook National University, 80, Daehak-ro, Buk-gu, Daegu, Republic of Korea, 41566

****Corresponding authors. Dementia Research Group, Korea Brain Research Institute, 61, Cheomdan-ro, Dong-gu, Daegu, Republic of Korea, 41062.

*****Corresponding authors. Dementia Research Group, Korea Brain Research Institute, 61, Cheomdan-ro, Dong-gu, Daegu, Republic of Korea, 41062.

E-mail address:

[kimchuna@kribb.re.kr](mailto:kimchuna@kribb.re.kr) (C. Kim), [sw_lee@korea.ac.kr](mailto:sw_lee@korea.ac.kr) (S-W. Lee), [srk75@knu.ac.kr](mailto:srk75@knu.ac.kr) (S.R. Kim), [nmy92@kbri.re.kr](mailto:nmy92@kbri.re.kr) (M. Nahm), [kim_jaekwang@kbri.re.kr](mailto:kim_jaekwang@kbri.re.kr) (J. Kim)

^1^These authors contributed equally to this work.

**This file contains the following Supplementary Information for Figures**

**Page 4-5:** Supplementary Figure 1. Establishment of a N2a neuronal cell model of TDP-43 overexpression.

**Page 6:** Supplementary Figure 2. Validation of knockdown efficiency of Trx1-specific siRNA.

**Page 7:** Supplementary Figure 3. Overexpressing Trx1 decreases insoluble TDP-43 levels in N2a-hTDP-43 cells.

**Page 8-9:** Supplementary Figure 4. TDP-43 neither interacts with Trx1 at the protein level nor affects the nuclear to cytoplasmic distribution of Trx1 mRNAs.

**Page 10:** Supplementary Figure 5. Establishment of a primary neuron model of TDP-43 overexpression.

**Supplementary Figure 1**

**Supplementary Figure 1. Establishment of a N2a neuronal cell model of TDP-43 overexpression.** N2a cells were transfected with human full-length TDP-43 WT. Cells transfected with control vectors served as negative controls. (A-B) Quantification of total TDP-43 expression following hTDP-43 overexpression in N2a cells. 48 h post-transfection, as indicated, total TDP-43 protein levels were analyzed by Western blot using an antibody recognizing both mouse and human TDP-43 (A). Total TDP-43 protein level was normalized to the corresponding GAPDH level and presented relative to the mean value of the control group (B) (n = 3, unpaired t-test). (C,D) Overexpression of hTDP-43 induces cytoplasmic TDP-43 aggregation. 48 h post-transfection, TDP-43 protein levels were analyzed in Triton X-100-soluble and -insoluble fractions by Western blot (C). Subcellular localization of TDP-43 was assessed by staining the cells with a TDP-43-specific antibody (D). Yellow arrows indicate cytoplasmic TDP-43 inclusions. Scale bars correspond to 25 µm. (E) Overexpression of hTDP-43 increases ROS levels in N2a cells. 48 h post-transfection, N2a cells were stained with CellROX Deep Red dye to detect ROS. Scale bars correspond to 25 µm. (F,G) TDP-43 overexpression does not induce stress granule formation in N2a cells. 48 h post-transfection, cells were stained with anti-G3BP1 antibody (F) or anti-TIA1 antibody (G), respectively. As a positive control for stress granule induction, cells were treated with 0.5 mM sodium arsenite (NaAsO₂) for 30 min prior to fixation. Green puncta (yellow arrowheads) represent stress granules. Nuclei were stained with DAPI. Scale bars correspond to 10 µm. Veh, vehicle.

**Supplementary Figure 2**

**Supplementary Figure 2. Validation of knockdown efficiency of Trx1-specific siRNA.** (A) N2a cells were transfected with 40 nM *Trx1*-specific siRNA (siTrx1) or negative control siRNA (siCon). 48 h post-transfection, endogenous *Trx1* mRNA levels were analyzed by qRT-PCR and normalized to the corresponding *Gapdh* levels (n = 3, unpaired *t*-test). (B,C) Endogenous Trx1 protein levels were assessed by immunostaining with a Trx1-specific antibody (B). Nuclei were stained with DAPI. The fluorescence intensities of Trx1 were quantified and normalized to the corresponding cell numbers (C) (n = 3, unpaired *t*-test). Scale bars correspond to 25 µm. All values are means ± SEM. * *p* < 0.05, ****p* < 0.001.

**Supplementary Figure 3**

**Supplementary Figure 3. Overexpressing Trx1 decreases insoluble TDP-43 levels in N2a-hTDP-43 cells.** 48 h post-transfection, as indicated, cell lysates were fractionated into Triton X-100-soluble and -insoluble fractions (A). TDP-43 protein levels were assessed by Western blot in Triton X-100-soluble (B) and -insoluble fractions (C) (n = 4, unpaired *t-*test). Soluble- and insoluble TDP-43 levels were normalized to the corresponding GAPDH and Ponceau S levels, respectively. Data are presented relative to the mean value of the control group (n = 4, unpaired *t-*test).

**Supplementary Figure 4**

**Supplementary Figure 4. TDP-43 neither interacts with Trx1 at the protein level nor affects the nuclear to cytoplasmic distribution of Trx1 mRNAs.** (A-D) TDP-43 does not interact with Trx1 protein. (A) Overexpressing TDP-43 does not affect the levels of endogenous *Trx1* mRNAs in N2a cells. 48 h post-transfection, endogenous mouse *Trx1* (*mTrx1)* mRNA levels were analyzed by qRT-PCR and were normalized to the corresponding *Gapdh* levels*.* Each level was presented as a relative fold change compared to control (n = 3, unpaired *t-*test). (B,C) TDP-43 protein does not interact with Trx1 protein. 48 h post-transfection, as indicated, Trx1 (B) or TDP-43 proteins (C) were immunoprecipitated using an anti-FLAG antibody. The protein–protein interaction between TDP-43 and Trx1 was assessed by Western blot. N2a cells transfected with control (Con) served as a negative control. HSP70 served as a positive control for TDP-43 interaction (C). (D) Proximity ligation assay (PLA) analysis reveals no detectable interaction between TDP-43 and Trx1 protein. 48 h post-transfection, PLA signals (red) were obtained as described in the Materials and Methods. HSP70 served as a positive control for TDP-43 interaction. The region outlined by the dashed box on the left is enlarged on the right. Nuclei were stained with DAPI (blue). Scale bars, 25 μm. (E, F) The nuclear-to-cytoplasmic ratio of *Trx1* mRNAs is not altered upon TDP-43 overexpression in N2a cells. 48 h post-transfection, cell lysates were fractionated into nuclear and cytoplasmic fractions as described in the Materials and Methods. GAPDH and Histone H3 were used as markers for the cytoplasmic and nuclear fraction, respectively (E). The nuclear-to-cytoplasmic ratio of *Trx1* mRNAs is presented as a relative fold change to control (F) (n = 3*,* unpaired *t*-test).

**Supplementary Figure 5**

**Supplementary Figure 5. Establishment of a primary neuron model of TDP-43 overexpression.** (A) Overexpression of TDP-43 induces cytoplasmic TDP-43 aggregation. Primary cortical neurons were transduced with AAV-hTDP-43 at DIV7 and stained with anti-TDP-43 and anti-MAP2 antibodies at DIV14. Nuclei are outlined by dashed circles. Scale bars correspond to 10 μm. (B,C) Neurite outgrowth is significantly inhibited by TDP-43 overexpression in primary cortical neurons. Neurons were stained with anti-TUBB3 (red) and anti-NeuN (green) antibodies at DIV14. Nuclei were stained with DAPI (blue) (B). The total neurite lengths were analyzed using Metamorph software and normalized to the corresponding cell numbers (C) (n = 5, *unpaired t-test*). All values are means ± SEM. ****p* < 0.001. Scale bars correspond to 100 μm. (D) Validation of Trx1 expression by AAV-hTrx1-Myc-FLAG virus transduction. Primary cortical neurons were transduced with AAV-hTrx1-Myc-FLAG viruses at DIV7 and stained with anti-Myc (green) and anti-TUBB3 (red) antibodies at DIV14. Nuclei were stained with DAPI (blue). Scale bars correspond to 50 μm.
